# 

**Published:** 1875-12

**Authors:** 


THE POCKET

### DENTAL REGISTER.

Every dentist with anything like a full practice, and who cares to conduct business in a systematic manner, must have a book in which to record his dental operations.

The large "Registers" that have been used for that purpose, are excellent in their place, and may still be used in connection with such a book as this, if desired; but they are open to several important objections.

Many dentists using them have doubtless experienced the inconvenience of going to the desk after each patient's sitting, to make a record, and have preferred trusting to memory till after office hours. By that time, the exact tooth ope ated upon has often been forgotten; and sometimes a certain operation has slipped from the mind altogether.

The fillings put in to-day are marked on the same diagram with those inserted a month or a year ago; consequently, after a little while, it is impossible to distinguish between old and recent work.

The space reserved for each account being so large, one is unwilling to spoil a blank to record trifling services, such as extracting or treating a tooth for a patient whem he may never see again; although it often happens afterward that he would be glad to have a knowledge of the service.

In recording operations on the temporary teeth, it is very unsatisfactory to cross out the twelve back teeth of the permanent set, and imagine the bicuspids to be temporary molars. In such cases, much confusion generally follows in the course of a few years.

This POCKET REGISTER is presented with the belief that it is free from these objec. tionable features.

Each operation may be recorded with pen or pencil, right at the chair, in almost the same time that an appointment is made for another sitting.

Every operation bears its own date, and cannot be taken for a previous or later one.

The space for accounts is so economically used, that one will not hesitate to record the most trivial services. It consists of 224 pages, arranged as follows:

120 pages for small account , ten to the page, with blanks for date, name, residence, operation, debit, and credit. Each account is also furnished with a diagram of the teeth for marking operations performed.
24 pages for medium accounts, two to the page, with blanks and diagram, as above.
16 pages for large accounts, one to the page, with blanks and diagram.
16 pages with blanks and diagrams expressly arranged for operations on the temporary teeth, ten accounts to the page.
32 pages for appointments for 64 weeks. These are without date, and may, therefore, be used for any time,
16 pages for index and memoranda.

It will be seen that the "Pocket Register" combines Account-book, Record of Operations on the Permanent and Temporary Teeth, Appointment-book, and Memoranda; and that the whole is offered for about the price of an ordinary Engagement book.

It is printed on fine writing paper, bound handsomely in leather, with tuck, or in English cloth, and is of a suitable size for carrying in the breast pocket.

— PRICE. —

*In Leather, with Tuck.* .......... $1 50
*In English Cloth,* .......... 1 25

Sent post-paid, on receipt of price. For sale, wholesale and retail, by

**For Sale by SPENCER, CROCKER & CO.**

AMERICAN DENTAL ASSOCIATION—Meets on the first Tuesday in Aug., 1875, at Niagara Falls. *Pres.* M. S. Dean, Chicago; *Cor. Sec.* Geo. L, Filed, Detroit; *Rec. Sec.* C. Stodard Smith, Springfield, Ill.

*American Dental Convention* Meets at Long Branch, second Tuesday in August, 1875. *Pres.* B. F. Coy, Baltimore; *Cor. Sec.* D. Roberts, Philadelphia, Pa.; *Rec. Sec.* A. Tees, Philadelphia, Pa.

*List of Societies Represented in the American Dental Association.*

*American Academy of Dental Science* Meets at Boston, Mass. *Pres.* Joshua Tucker M. D., of Boston; *Sec.* W. L. Tucker, of Boston; *Cor. Sec.*, E. N. Harris.

*Brooklyn Dental Society* Meets semi-monthly. *Pres.* M. E. Elmandorf; *Rec. Sec.* C. P. Crandell; *Cor. Sec.* A. N. Chapman.

*Buffalo Dental Society* Meets second Monday of each month at Buffalo. *Pres.* S. A. FREEMAN; *Rec. Sec.* G. C. DABOLL.

*Boston Dental Institute*—Meets first Tuesday of each month. *Pres.* D. G. WILLIAMS, Buffalo; *Cor. Sec.* D. S. Dickerman.

*California State Dental Association* Meets annually in June. *Pres.* C. C. Knowles, San Francisco; *Rec. Sec.* H. J. Plomteaux, Woodland.

*Cincinnati Dental Society*—Meets Semi-monthly at Cincinnati. *Pres.* H. R. SMITH; *Rec. Sec.* D. C. HAWXHURST.

*Chicago Dental Society* Meets monthly at Chicago. *Pres.* M. S. Dean; *Rec. Sec.* Edgar D. Swain.

*Connecticut Valley Dental Association.* Meets semi-annually, second Tuesday of June and October. *Pres.* H. M. Miller, Westfield, Mass.; *Rec. Sec.* L. C. Taylor, of Holyoke, Mass.

*Connecticut State Dental Association.* *Pres.* R. C. Dunham, New Britain; *Rec. Sec.* D. E. Lane, Hartford.

*Charleston Dental Association*—Meets second Tuesday of every month; *Pres.* T. F. Chupein; *Sec. and Treas.* B. A. Muckenfuss.

*Delaware Dental Association*—Meets semi-annually. *Pres.* E. W. Haines, Newark; *Sec.* C. R. Jeffries, Wilmington.

*Eastern Indiana Dental Association.*—Meets semi-annually, first Tuesday in May and last Tuesday in October; *Pres.* J. K. Jameson, Connersville; *Sec.* J. W. White, Knightstown, Ind. (Place of meeting fixed by appointment.)

*Eastern Ohio Dental Association*—Meets on Tuesday, August 26th, 1873, at Alliance; *Pres.* A. J. Douds; *Sec.* J. M. Porter.

*East Tennessee Dental Association*—Meets annually at Knoxville, Tenn. *Pres.* Wm. W. F. Fowler; *Rec. Sec.* S. M. Prothro.

*Forest City Society of Dental Surgeons*—*Pres.* B. Strickland, Cleveland, *Rec. Sec.* H. L. Ambler, Cleveland; *Cor. Sec.* Chas. Buffett, Cleveland.

*Georgia State Dental Society* Next meeting at Atlanta, July 1873. *Pres.* F. Y. Clark; *Cor. Sec.* J. P. H. Brown, Augusta.

*Harris Dental Association* Meets quarterly on the first Thursday in February, May, August, November. *Pres.* J. A. Martin, Strasburg; *Sec.* Wm. N. Amer, Lancaster,

*Hudson River Association of Dental Surgeons* Meets on the second Wednesdays of May, September and January. *Pres.* L. S. Straw, Newburg, N. Y; *Rec. Sec.* T. W. DuBois, Poughkeepsie, N. Y.

*Hudson Valley Dental Association* Meets monthly at Troy, N. Y. *Pres.* L. C. Wheeler, *Rec. Sec.* S. G. Andres; *Cor. Sec.* S. D. French.

*Illinois State Dental Society* Meets annually. *Pres.* G. S. Miles, Jerseyville, *Sec.* C. R. E. Koch, Chicago. Meets second Tuesday in May, 1875, at Ottowa.

*Indiana State Dental Society*—Meets next year at Indianapolis, on the first Tuesday of June. *Pres.* M. Wells, Indianapolis; *Cor. and Rec. Sec.* P. McDonald, Goshen.

*Iowa State Dental Society*—Meets annually. Next meeting at Davenport, on the second Tuesday of May. *Pres.* L. C. Ingersoll, Keokuk. *Rec. Sec.* E. P. White, of Davenport; *Cor. Sec.* A. J. Redrich, Sioux City.

*Kansas State Dental Society* Meets annually. Next meeting at Leavanworth, first Wednesday in May, 1875. *Pres.* L. C. Wasson; *Sec.* J. D Patterson.

*Kansas and Missouri Valley Dental Association*—Meets first and third Tuesdays of erch month. *Pres.* J. R. Stark; *Sec.* H. C. Massie, Kansas City, Mo.

*Kentucky State Dental Association* Meets annually first Tuesday in June, next meeting in Lexington. *Pres.*, B. O. Doyle; *Sec.*, Chas. E Dunn; both of Louisville.

*Lake Erie Dental Association* Meets Annually. *Pres.* C. B. Ansart, Oil City, Pa. *Sec.* C. D. Elliot, Franklin, Pa. Next meeting first Tuesday of May, 1874.

*Mad River Dental Association* Meets at Dayton on the first Tuesdays in April and October. *Pres.* S. B. Tizzard, Dayton; *Sec.* A. J. Grosvenor, Troy.

*Maine Dental Society*—Meets in Aug., at Thomaston. *Pres.* E. J. Roberts, Augusta; *Sec.* C. E. Hussey, Biddeford.

*Maryland Association of Dentists* Meets the last Thursday of every month. *Pres.* J. B. Bean; *Sec.* S. M. Field.

*Massachusetts Dental Society*—Meets monthly. *Pres.* J. H. Batchelder, of Salem *Sec.* C. Wilson; *Cor. Sec.* L. D. Shepard.

*Michigan State Dental Association*—Meets annually second Tuesday of Oct., at Ypsilanti. *Pres.* D. C. Hawxhurst, Ann Arbor; *Rec. & Cor. Sec.* T. R. Perry, Grand Rapids

*Michigan Central Dental Association.* Meets annually at Jackson, Secon Wednesday of January, 1873. *Pres.* C. C. Jenkins, Anu Arbor, *Sec.* W. H. Darrance, Jackson.

*Mississippi Valley Dental Association* Meets annually at Cincinnati, on first Wednesday in March, 1874. *Pres.* S. B. Brown, Ft. Wayne Ind.; *Rec. Sec.* F. Hunter Cin.

*Minnesota Dental Associrtion* Meets annually. Next meeting at Faribault, last Wednesday in June, 1874. *Pres.* J. A. Bowman, Minneapolis; *Sec.* C. D. Montreville, St. Paul.

*Missouri Dental Association* Meets on the first Tuesday of June, in St. Louis. *Pres.* C. W. Rivers, St. Louis; *Sec.* W. H. Evans, St. Louis.

*Missouri Valley Dental Association** Meets on the fourth Tuesday in July, in Nebraska City. *Pres.* A. S. Billings, Omaha, Neb.; *Sec. and Treas.* J. F. Sanborn, Tabor, Iowa.

*Merrimac Valley Dental Association* Meets semi-annually first Tuesday of Oct. and May.* *Pres.* L. H. Kidder, Mass; *Rec. Sec.* A. M. Dudley, of Lowell; *Cor. Sec.* D. T. Porter, Lawrence, Mass.

*Memphis Dental Society*—Meets on the first Tuesday of each month. *Pres.* W. M. Arrington; *Sec.* V. F. Elliott; *Treas.* C. L. Harris.

*Nashville Dental ssociation*--Meets on the second Tuesday of each month. *Pres.* J. C. Ross; *Sec.* R. R. Freeman; *Cor. Sec.* S. J. Cobb.

*New Jersey State Dental Society*—Meets annually. Next meeting at Long Branch, on the first Tuesday in July, 1875. *Pres.* Geo. C. Brown, Mount Holly; *Sec.* J. W. Scarborough, Lambertville.

*New Haven Dental Society*--Meets on the first Wednesday of each month at New Haven. *Pres.* J. T. Metcalf, *Rec and Cor. Sec.* C. L. Smith, of New Haven.

*New Orleans Dental Society* Meets monthly. *Pres.* ——————; *Rec. Sec.* A. F McLane.

*Northern Ohio Dental Association.* Meets annually, second Tuesday of June, 1873, at Put-in Bay. *Pres.* J. A. Robinson, Cleveland; *Rec. Sec.* A. F. Price, Fremont.

*Northern Iowa Dental Association*—Meets annually, on the second Tuesday in June, 1873. Next meeting at Waterloo; *Pres.* J. N. Abbott, Lancaster, *Rec. Sec.* A. V. Eaton, Anamosa; *Cor. Sec.* A. R. Mason, Waterloo.

*Ohio College Dental Association*—Meets annually Feb. 25. at Cincinnati. *Pres.* Jas. Leslie, Cin'ti.; *Rec. Sec.* H. A. Smith, of Cincinnati.

*Ohio State Dental Association*--Meets annually at Columbus. Next meeting, first Tuesday of December. *Pres.* C. R. Butler; *Rec. Sec.* F. A. Hunter, Cincinnati; *Cor. Sec.* C. R. Butler, Cleveland, O.

*Odontographic Society of Pennsylvania*—Meets monthly in Philadelphia. Next meeting, Wednesday, Sept. 1st. *Pres.* C. T. Stellwagen; *Rec. Sec.* E. L. Hewitt.

*Odontological Society of New York City* Meets the second Tuesday of each monh. *Pres.* A. L. Northrup; *Rec. Sec.* Wm. Jarvie, Jr.

*Old Colony Dental Association* Meets quarterly.* *Pres.* C. G. Davis; *Rec. Sec.* L. W. Puffer, North Bridgewater, Mass; *Cor. Sec.* N. C. Fowler, Yarmouth, Mass.

*Ohio Valley Dental Association* Meets annually, in N. E. Ky; this year in Paris, Ky. Tuesday, Sept. 9th.

*Oregon State Dental Society* Meets annually. *Pres.* J. H. Hatch, Portland; *Rec. Sec.* J. R. Cardwell, Portland.

*Pennsylvania State Dental Society* Meets next at Wilkesbarre, second Tuesday of July, 1874. *Pres.* G. W. Klump, Williamsport; *Rec. Sec.* R. H. Moffit, Harrisburg *Cor. Sec.* S. Welchens, Lancaster.

*Poughkeepsie Dental Society* Meets monthly. *Pres.* J. H. Mann; *Sec.* H. F. Clark

*Pennsylvania Association of Dental Surgery* Meets monthly in Philadelphia. *Pres.* Spencer Roberts; *Sec.* E. R. Pettit.

*San Francisco Dental Association* Meets monthly. *Pres.* C. C. Knowles; *Cor. Sec.* H. E. Knox; *Rec. Sec.* Wm. Dutch.

*St. Louis Dental Society* Meets monthly. *Pres.* W. H. Morrison; *Sec.* A. H. Fuller.

*Susquehanna Dental Association* Meets annually.* *Pres.* C. S. Beck: *Rec. Sec.* W. H. Nertz. Meets May, 1875, at Scranton.

*Society of Dental Surgeons of the City of New York* Meets semi-monthly in New York. *Pres.* B. F. Franklin: *Rec. Sec.* J. S. Latimer; *Cor. Sec.* E. H. Burras.

**Southern Dental Association* Meets annually. Next meeting at Memphis, Tenn., at time of Mardi-Gras. *Pres.* J. R. Walker, New Orleans, La.; *Rec. Sec.* J. F Thompson Va.

*South Carolina State Dental Association* Meets semi-annually in May and Nov. *Pres.* T. F. Chupein, Charleston; *Sec.* J. W. Norwood, Greenville; *Cor. Sec.* C. C. Patrick, Charleston, S. C. Next meeting at Columbia.

*Tennessee Dental Association* Meets semi-annually. Next meeting in Nashville, on the 4th Wednesday in June. 1875. *Pres.* E. S. Chisholm; *Sec.* J. S. King; *Cor. Sec.* S. J. Cobb.

*Virginia State Dental Association* Meets in Richmond, first Thursday in Nov. of each year. *Pres.* H. M. Grant, Abington: *Sec.* G. F. Kersee, Richmond.

*Washington, City Dental Society* Meets annually. *Pres.* R. F. Hunt, *Sec.* Dr. Evans.

*Wabash Valley Dental Association* Meets annually. Next meeting at Wayne, third Tuesday in March, 1872. *Pres.* A. B. Cunningham, Attica: *Sec.* S. B. Brown, Fort Wayne.

* *Wisconsin State Dental Association* Meets semi-annually. Next meeting at Watertown. second Tuesday of January, 1872. *Pres.* Edgar Palmer, Le Crook *Sec.* C. C. Chittenden, Madison.

**Place of meeting fixed by appointment*

*New York State Dental Society*--Meets at Albany on the last Wednesday of June next. *Pres.* W. C. Barret, Warsaw, N. Y.; *Cor. Sec.* W. S. Elliott, Goshen; *Rec. Sec.* Charles Barnes, Syracuse.

### District Dental Societies of the State of New York.

*First District* Meets second and fourth Wednesday evenings of each month. Annual meeting in New York, first Tuesday in June. *Pres.* R. M, Gage; *Rec. Sec.* W. E. Hoag, 331 Madison ave., New York.

*Second District.* Annual meeting in Brooklyn, first Monday in April. *Pres.* W. S. Elliott; *Rec. Sec.* M. E. Elmandorf. Brooklyn; *Cor. Sec.* F. W. Dalbare.

*Third District.* Meets semi-annually on second Tuesday in January and July. Annual meeting at Albany on second Tuesday in January. *Pres.* J. C. Austin. Albany; *Rec. Sec.* S. J. Andress, Troy; *Cor. Sec.* D. H. Young, Troy.

*Fourth District.* Meets semi-annually. Meets annually at Saratoga on second Tuesday in June. *Pres.* C, A. Elmore, Ft. Edward; *Rec. Sec.* E. S. Pearsall, Saratoga.

*Fifth District.* Meets semi-annually. Annual meeting at Syracuse. second Tuesday in June. *Pres.* J. D. Huntington, Watertown; *Rec. Sec.* F. D. Nellis, Syracuse; *Cor. Sec.* J. S. Marshall, Syracuse.

*Sixth District.* Meets semi-annually. Annual meeting at Binghampton on second Tuesday in June. *Pres.* Frank B. Darby, Oswego; *Rec. Sec.* F. E. Perry, Norwich; *Cor. Sec.* A. M. Holmes, Morrisville.

*Seventh District.* Meets semi-annually. Annual meeting at Rochester first Tuesday in June. *Pres.* H. S. Miller; *Rec. Sec.* J. E. Line, Rochester.

*Eighth District.* Meets semi-annually. Annual on second Tuesday in May. *Pres* G. C. Daboll, *Rec. Sec.* S. A. Freeman, *Cor. Sec.* T. G. Lewis, Buffalo.

*Buffalo Dental Association.* Meets on second Tuesday evening of each month. Annual meeting in June. *Pres.* S. A. Freeman; *Sec.* W. H. Barrows.

The Ohio State Board of Dental Examiners will meet in Columbus on the first Tuesday of Dec. 1875, at 10 o'clock. *Pres.* J. Taft; *Sec.* W. P. Horton.

### List of Exchanges.

ST. LOUIS MEDICAL AND SURGICAL JOURNAL.
BOSTON MEDICAL AND SURGICAL JOURNAL.
THE PACIFIC MEDICAL AND SURGICAL JOURNAL, SAN FRANCISCO.
PHIDADELPHIA MEDICAL AND SURGICAL JOURNAL.
CINCINNATI MEDICAL ADVANCE.
CINCINNATI LANCET AND OBSERVER.
DENTAL COSMOS, PHILADELPHIA,
LADIES' REPOSITORY, CINCINNATI.
HALL'S JOURNAL OF HEALTH, N. Y.
CHICAGO MEDICAL EXAMINER,
THE DETROIT REVIEW OF MEDICINE AND PHARMACY.
MEDICAL RECORD, N. Y.
NASHVILLE JOURNAL OF MEDICINE AND SURGERY.
AMERICAN JOURNAL OF DENTAL SCIENCE, BALTIMORE.
THE UNITED STATES MEDICAL AND SURGICAL JOURNAL, CHICAGO
NEW ORLEANS JOURNAL OF MEDICINE,
AMERICAN AGRICULTURIST, N. Y.
BOSTON JOURNAL OF CHEMISTRY.
JOURNAL OF APPLIED CHEMISTRY, N. Y.
CANADA JOURNAL OF DENTAL SCIENCE, MONTREAL.
INDIANA JOURNAL OF MEDICINE,
MEDICAL AND SURGICAL REPORTER, SALEM, OREGON
MISSOURI DENTAL JOURNAL, ST. LOUIS.
ECLECTIC MEDICAL JOURNAL, CINCINNATI.

AMERICAN DENTAL ASSOCIATION—Meets on the first Tuesday in Aug., 1875, at Niagara Falls. *Pres.* M. S. Dean, Chicago; *Cor. Sec.* Geo. L, Field, Detroit; *Rec. Sec.* C. Stodard Smith, Springfield, Ill.

*American Dental Convention* Meets at Long Branch, second Tuesday in August, 1875. *Pres.* B. F. Coy, Baltimore: *Cor. Sec.* D. Roberts, Philadelphia, Pa.; *Rec. Sec.* A. Tees, Philadelphia, Pa.

*List of Societies Represented in the American Dental Association.*

*American Academy of Dental Science* Meets at Boston, Mass. *Pres.* Joshua Tucker M. D., of Boston ; *Sec.* W. L. Tucker, of Boston ; *Cor. Sec.*, E. N. Harris.

*Brooklyn Dental Society* Meets semi-monthly. *Pres.* M. E. Elmandorf; *Rec. Sec.* C. P. Crandell ; *Cor. Sec.* A. N. Chapman.

*Buffalo Dental Society* Meets second Monday of each month at Buffalo. *Pres.* S. A. FREEMAN ; *Rec. Sec.* G. C. DABOLL.

*Boston Dental Institute*—Meets first Tuesday of each month. *Pres.* D. G. WILLIAMS, Buffalo; *Cor. Sec.* D. S. Dickerman.

*California State Dental Association* Meets annually in June. *Pres.* C. C. Knowles, San Francisco ; *Rec. Sec.* H. J. Plomteaux, Woodland.

*Cincinnati Dental Society*—Meets Semi-monthly at Cincinnati. *Pres.* H. R. SMITH ; *Rec. Sec.* D. C. HAWXHURST.

*Chicago Dental Society* Meets monthly at Chicago. *Pres.* M. S. Dean ; *Rec. Sec.* Edgar D. Swain.

*Connecticut Valley Dental Association.* Meets semi-annually, second Tuesday of June and October. *Pres.* H. M. Miller, Westfield, Mass.; *Rec. Sec.* L. C. Taylor, of Holyoke, Mass.

*Connecticut State Dental Association.* *Pres.* R. C. Dunham, New Britain ; *Rec. Sec.* D. E. Lane, Hartford.

*Charleston Dental Association*—Meets second Tuesday of every month ; *Pres.* T. F. Chupein ; *Sec. and Treas.* B. A. Muckenfuss.

*Delaware Dental Association*—Meets semi-annually. *Pres.* E. W. Haines, Newark ; *Sec.* C. R. Jeffries, Wilmington.

*Eastern Indiana Dental Association.*—Meets semi-annually, first Tuesday in May and last Tuesday in October; *Pres.* J. K. Jameson, Connersville; *Sec.* J. W. White, Knightstown, Ind. (Place of meeting fixed by appointment.)

*Eastern Ohio Dental Association*—Meets on Tuesday, August 26th, 1873, at Alliance ; *Pres.* A. J. Douds ; *Sec.* J. M. Porter.

*East Tennessee Dental Association*—Meets annually at Knoxville, Tenn. *Pres.* Wm. W. F. Fowler; *Rec. Sec.* S. M. Prothro.

*Forest City Society of Dental Surgeons*—*Pres.* B. Strickland, Cleveland, *Rec. Sec.* H. L. Ambler, Cleveland ; *Cor. Sec.* Chas. Buffett, Cleveland.

*Georgia State Dental Society* Next meeting at Atlanta, July 1873. *Pres.* F. Y. Clark ; *Cor. Sec.* J. P. H. Brown, Augusta.

*Harris Dental Association* Meets quarterly on the first Thursday in February, May. August, November. *Pres.* J. A. Martin, Strasburg ; *Sec.* Wm. N. Amer, Lancaster,

*Hudson River Association of Dental Surgeons* Meets on the second Wednesdays of May, September and January. *Pres.* L. S. Straw, Newburg, N. Y ; *Rec. Sec.* T. W. DuBois, Poughkeepsie, N. Y.

*Hudson Valley Dental Association* Meets monthly at Troy, N. Y. *Pres.* L. C. Wheeler, *Rec. Sec.* S. G. Andres ; *Cor. Sec.* S. D. French.

*Illinois State Dental Society* Meets annually. *Pres.* G. S. Miles, Jerseyville, *Sec.* C. R. E. Koch, Chicago. Meets second Tuesday in May, 1875, at Ottowa.

*Indiana State Dental Society*—Meets next year at Indianapolis, on the first Tuesday of June. *Pres.* M. Wells, Indianapolis ; *Cor. and Rec. Sec.* P. McDonald, Goshen.

*Iowa State Dental Society*—Meets annually. Next meeting at Davenport, on the second Tuesday of May. *Pres.* L. C. Ingersoll, Keokuk. *Rec. Sec.* E. P. White, of Davenport ; *Cor. Sec.* A. J. Redrich, Sioux City.

*Kansas State Dental Society* Meets annually. Next meeting at Leavanworth, first Wednesday in May, 1875. *Pres.* L. C. Wasson; *Sec.* J. D Patterson.

*Kansas and Missouri Valley Dental Association*—Meets first and third Tuesdays of erch month. *Pres.* J. R. Stark ; *Sec.* H. C. Massie, Kansas City, Mo.

*Kentucky State Dental Association* Meets annually first Tuesday in June, next meeting in Lexington. *Pres.*, B O. Doyle; *Sec.*, Chas. E Dunn; both of Louisville.

*Lake Erie Dental Association* Meets Annually. *Pres.* C. B. Ansart, Oil City, Pa. *Sec.* C. D. Elliot, Franklin, Pa. Next meeting first Tuesday of May, 1874.

*Mad River Dental Association* Meets at Dayton on the first Tuesdays in April and October. *Pres.* S. B. Tizzard, Dayton ; *Sec.* A. J. Grosvenor, Troy.

*Maine Dental Society*—Meets in Aug., at Thomaston. *Pres.* E. J. Roberts, Augusta ; *Sec.* C. E. Hussey, Biddeford.

*Maryland Association of Dentists* Meets the last Thursday of every month. *Pres.* J. B. Bean ; *Sec.* S. M. Field.

*Massachusetts Dental Society*—Meets monthly. *Pres.* J. H. Batchelder, of Salem *Sec.* C. Wilson ; *Cor. Sec.* L. D. Shepard.

*Michigan State Dental Association*—Meets annually second Tuesday of Oct., at Ypsilanti. *Pres.* D. C. Hawxhurst, Ann Arbor; *Rec. & Cor. Sec.* T. R. Perry, Grand Rapids

*Michigan Central Dental Association.* Meets annually at Jackson, Secon Wednesday of January, 1873. *Pres.* C. C. Jenkins, Anu Arbor, *Sec.* W. H. Darrance, Jackson.

*Mississippi Valley Dental Association* Meets annually at Cincinnati, on first Wednesday in March, 1874. *Pres.* S. B. Brown, Ft. Wayne Ind.; *Rec. Sec.* F. Hunter Cin.

*Minnesota Dental Associrtion* Meets annually. Next meeting at Faribault, last Wednesday in June, 1874. *Pres.* J. A. Bowman, Minneapolis; *Sec.* C. D. Montreville, St. Paul.

*Missouri Dental Association* Meets on the first Tuesday of June, in St. Louis. *Pres.* C. W. Rivers, St. Louis; *Sec.* W. H. Evans, St. Louis.

*Missouri Valley Dental Association** Meets on the fourth Tuesday in July, in Nebraska City. *Pres.* A. S. Billings, Omaha, Neb.; *Sec. and Treas.* J. F. Sanborn, Tabor, Iowa.

*Merrimac Valley Dental Association* Meets semi-annually first Tuesday of Oct. and May.* *Pres.* L. H. Kidder, Mass; *Rec. Sec.* A. M. Dudley, of Lowell; *Cor. Sec.* D. T. Porter, Lawrence, Mass.

*Memphis Dental Society*—Meets on the first Tuesday of each month. *Pres.* W. M. Arrington; *Sec.* V. F. Elliott; *Treas.* C. L. Harris.

*Nashville Dental ssociation*—Meets on the second Tuesday of each month. *Pres.* J. C. Ross; *Sec.* R. R. Freeman; *Cor. Sec.* S. J. Cobb.

*New Jersey State Dental Society*—Meets annually. Next meeting at Long Branch, on the first Tuesday in July, 1875. *Pres.* Geo. C. Brown, Mount Holly; *Sec.* J. W. Scarborough, Lambertville.

*New Haven Dental Society*—Meets on the first Wednesday of each month at New Haven. *Pres.* J. T. Metcalf, *Rec and Cor. Sec.* C. L. Smith, of New Haven.

*New Orleans Dental Society* Meets monthly. *Pres.* ————————; *Rec. Sec.* A. F McLane.

*Northern Ohio Dental Association.* Meets annually, second Tuesday of June, 1873, at Put-in Bay. *Pres.* J. A. Robinson, Cleveland; *Rec. Sec.* A. F. Price, Fremont.

*Northern Iowa Dental Association*—Meets annually, on the second Tuesday in June, 1873. Next meeting at Waterloo; *Pres.* J. N. Abbott, Lancaster, *Rec. Sec.* A. V. Eaton, Anamosa; *Cor. Sec.* A. R. Mason, Waterloo.

*Ohio College Dental Association*—Meets annually Feb. 25. at Cincinnati. *Pres.* Jas. Leslie, Cin'ti.; *Rec. Sec.* H. A. Smith, of Cincinnati.

*Ohio State Dental Association*—Meets annually at Columbus. Next meeting, first Tuesday of December. *Pres.* C. R. Butler; *Rec. Sec.* F. A. Hunter, Cincinnati; *Cor. Sec.* C. R. Butler, Cleveland, O.

*Odontographic Society of Pennsylvania*—Meets monthly in Philadelphia. Next meeting, Wednesday, Sept. 1st. *Pres.* C. T. Stellwagen; *Rec. Sec.* E. L. Hewitt.

*Odontological Society of New York City* Meets the second Tuesday of each monh. *Pres.* A. L. Northrup; *Rec. Sec.* Wm. Jarvie, Jr.

*Old Colony Dental Association* Meets quarterly.* *Pres.* C. G. Davis; *Rec. Sec.* L. W. Puffer, North Bridgewater, Mass; *Cor. Sec.* N. C. Fowler, Yarmouth, Mass.

*Ohio Valley Dental Association* Meets annually, in N. E. Ky; this year in Paris, Ky. Tuesday, Sept. 9th.

*Oregon State Dental Society* Meets annually. *Pres.* J. H. Hatch, Portland; *Rec. Sec.* J. R. Cardwell, Portland.

*Pennsylvania State Dental Society* Meets next at Wilkesbarre, second Tuesday of July, 1874. *Pres.* G. W. Klump, Williamsport; *Rec. Sec.* R. H. Moffit, Harrisburg *Cor. Sec.* S. Welchens, Lancaster.

*Poughkeepsie Dental Society* Meets monthly. *Pres.* J. H. Mann; *Sec.* H. F. Clark

*Pennsylvania Association of Dental Surgery* Meets monthly in Philadelphia. *Pres* Spencer Roberts; *Sec.* E. R. Pettit.

*San Francisco Dental Association* Meets monthly. *Pres.* C. C. Knowles; *Cor. Sec.* H. E. Knox; *Rec. Sec.* Wm. Dutch.

*St. Louis Dental Society* Meets monthly. *Pres.* W. H. Morrison; *Sec.* A. H. Fuller.

*Susquehanna Dental Association* Meets annually.* *Pres.* C. S. Beck: *Rec. Sec.* W. H. Nertz. Meets May, 1875, at Scranton.

*Society of Dental Surgeons of the City of New York* Meets semi-monthly in New York. *Pres.* B. F. Franklin: *Rec. Sec.* J. S. Latimer; *Cor. Sec.* E. H. Burras.

**Southern Dental Association* Meets annually. Next meeting at Memphis, Tenn., at time of Mardi-Gras. *Pres.* J. R. Walker, New Orleans, La.; *Rec. Sec.* J. F Thompson Va.

*South Carolina State Dental Association* Meets semi-annually in May and Nov. *Pres.* T. F. Chupein, Charleston; *Sec.* J. W. Norwood, Greenville; *Cor. Sec.* C. C. Patrick, Charleston, S. C. Next meeting at Columbia.

*Tennessee Dental Association* Meets semi-annually. Next meeting in Nashville, on the 4th Wednesday in June. 1875. *Pres.* E. S. Chisholm; *Sec.* J. S. King; *Cor. Sec.* S. J. Cobb.

*Virginia State Dental Association* Meets in Richmond, first Thursday in Nov. of each year. *Pres.* H. M. Grant, Abington: *Sec.* G. F. Kersee, Richmond,

*Washington, City Dental Society* Meets annually. *Pres.* R. F. Hunt, *Sec.* Dr. Evans.

*Wabash Valley Dental Association* Meets annually. Next meeting at Wayne, third Tuesday in March, 1872. *Pres.* A. B. Cunningham, Attica: *Sec,* S. B. Brown, Fort Wayne.

* *Wisconsin State Dental Association* Meets semi-annually. Next meeting at Watertown. second Tuesday of January, 1872, *Pres.* Edgar Palmer, Le Crook *Sec.* C. C. Chittenden, Madison.

**Place of meeting fixed by appointment*

*New York State Dental Society*--Meets at Albany on the last Wednesday of June next. *Pres.* W. C. Barret, Warsaw, N. Y.; *Cor. Sec.* W. S. Elliott, Goshen; *Rec. Sec.* Charles Barnes, Syracuse.

### District Dental Societies of the State of New York.

*First District* Meets second and fourth Wednesday evenings of each month. Annual meeting in New York, first Tuesday in June. *Pres.* R. M, Gage; *Rec. Sec.* W. E. Hoag, 331 Madison ave., New York.

*Second District.* Annual meeting in Brooklyn, first Monday in April. *Pres.* W. S. Elliott; *Rec. Sec.* M. E. Elmandorf. Brooklyn; *Cor. Sec.* F. W. Dalbare.

*Third District.* Meets semi-annually on second Tuesday in January and July. Annual meeting at Albany on second Tuesday in January. *Pres.* J. C. Austin, Albany; *Rec. Sec.* S. J. Andress, Troy; *Cor. Sec.* D. H. Young, Troy.

*Fourth District.* Meets semi-annually. Meets annually at Saratoga on second Tuesday in June. *Pres.* C, A. Elmore, Ft. Edward; *Rec. Sec.* E. S. Pearsall, Saratoga.

*Fifth District.* Meets semi-annually. Annual meeting at Syracuse. second Tuesday in June. *Pres.* J. D. Huntington, Watertown; *Rec. Sec.* F. D. Nellis, Syracuse; *Cor. Sec.* J. S. Marshall, Syracuse.

*Sixth District.* Meets semi-annually. Annual meeting at Binghampton on second Tuesday in June. *Pres.* Frank B. Darby, Oswego; *Rec. Sec.* F. E. Perry, Norwich; *Cor. Sec.* A. M. Holmes, Morrisville.

*Seventh District.* Meets semi-annually. Annual meeting at Rochester first Tuesday in June. *Pres.* H. S. Miller; *Rec. Sec.* J. E. Line, Rochester.

*Eighth District.* Meets semi-annually. Annual on second Tuesday in May. *Pres* G. C. Daboll, *Rec. Sec.* S: A. Freeman, *Cor. Sec.* T. G. Lewis, Buffalo.

*Buffalo Dental Association.* Meets on second Tuesday evening of each month. Annual meeting in June. *Pres.* S. A. Freeman; *Sec.* W. H. Barrows.

The Ohio State Board of Dental Examiners will meet in Columbus on the first Tuesday of Dec. 1875, at 10 o'clock. *Pres.* J. Taft; *Sec.* W. P. Horton.

### List of Exchanges.

ST. LOUIS MEDICAL AND SURGICAL JOURNAL.
BOSTON MEDICAL AND SURGICAL JOURNAL.
THE PACIFIC MEDICAL AND SURGICAL JOURNAL, SAN FRANCISCO.
PHIDADELPHIA MEDICAL AND SURGICAL JOURNAL.
CINCINNATI MEDICAL ADVANCE.
CINCINNATI LANCET AND OBSERVER.
DENTAL COSMOS, PHILADELPHIA,
LADIES' REPOSITORY, CINCINNATI.
HALL'S JOURNAL OF HEALTH, N. Y.
CHICAGO MEDICAL EXAMINER,
THE DETROIT REVIEW OF MEDICINE AND PHARMACY.
MEDICAL RECORD, N. Y.
NASHVILLE JOURNAL OF MEDICINE AND SURGERY.
AMERICAN JOURNAL OF DENTAL SCIENCE, BALTIMORE.
THE UNITED STATES MEDICAL AND SURGICAL JOURNAL, CHICAGO
NEW ORLEANS JOURNAL OF MEDICINE,
AMERICAN AGRICULTURIST, N. Y.
BOSTON JOURNAL OF CHEMISTRY.
JOURNAL OF APPLIED CHEMISTRY, N. Y.
CANADA JOURNAL OF DENTAL SCIENCE, MONTREAL.
INDIANA JOURNAL OF MEDICINE,
MEDICAL AND SURGICAL REPORTER, SALEM, OREGON
MISSOURI DENTAL JOURNAL, ST. LOUIS.
ECLECTIC MEDICAL JOURNAL, CINCINNATI.

### HARVARD UNIVERSITY.

### Dental Department.

### BOSTON, MASS. 1875–76.

#### FACULTY.

Charles William Eliot, L.L.D., *President.*
Oliver W. Holmes, M.D., Professor of Anatomy.
Henry J. Bigelow, M.D., Professor of Surgery and Clinical Surgery.
Thomas H. Chandler, D.M.D., Professor of Mechanical Dentistry.
George T. Moffatt, M.D., D.M.D., Professor of Operative Dentistry.
Luther D. Shepard, D.D.S., Adjunct Professor of Operative Dentistry.
Nathaniel W. Hawes, Assistant Professor of Operative Dentistry.
Edward S. Wood, M.D., Assistant Professor of Chemistry.
Henry P. Bowditch, M.D., Assistant Professor of Physiology.
William H. Rollins, D.M.D., Instructor in Dental Pathology.
Charles A. Brackett, D.M.D., Instructor in Dental Therapeutics.
Ira A. Salmon, D.D.S., University Lecturer on Operative Dentistry.
Charles B. Porter, M.D., Demonstrator of Practical Anatomy.
Charles Wilson, D.M.D., Demonstrator in Charge.
George F. Grant, D.M.D., Demonstrator of Mechanical Dentistry.

The Winter Session of the Eighth year of this School begins on the 30th of September and continues nineteen weeks. The Spring Session begins on Feb. 16th and ends June 29th. Two full Winter Courses are required for graduation. The spring session is simply to take the place of private pupilage, designed chiefly for those who wish to spend their whole three years of study in the school. Unsurpassed facilities for operative and mechanical practice are furnished by the infirmary of the Massachusetts General Hospital which is open throughout the year.

☞ The University Degree, D.M.D. (*Dentariæ Medicinæ Doctor*), is conferred upon those who fulfill the requirements.

#### FEES.

**Matriculation, $5.00. Spring Session, $50.00. Winter Session, $110.00.**
**For the Year, $150.00. Graduation, $30.00.**

From and after September 28, 1876, an entire change will be made in the scheme of Instruction. Instruction will be given by lectures, recitations, clinical teaching and practical exercises, uniformly distributed throughout the academic year, and the distinction of winter and summer sessions will thereafter be abolished.

The course of instruction will be progressive, extending over two years.

The regular examinations will be held in the following order, viz.: At the end of the first year, anatomy, including dissection, physiology and general chemistry. A certificate from the demonstrator of anatomy will be required of each student, that he has satisfactorily dissected the three parts of the body.

At the end of the second year, dental pathology, including a knowledge of gestation and diseases of woman so far as they affect the mouth and throat, dental materia medica and therapeutics, oral surgery and surgical pathology, operative and mechanical dentistry. The examinations in operative and mechanical dentistry will include actual operations and the preparation of specimens of mechanical dentistry.

Every candidate must be twenty-one years of age, and of good moral character; he must give evidence of having studied medicine or dentistry three full years; he must have spent at least one continuous year at this school, have presented a satisfactory thesis and have passed all the required examinations.

#### FEES UNDER THE NEW PLAN.

There shall be no fees for matriculation, for the diploma, nor for the demonstrators. For the first year a student is a member of the school, the fee shall be $200; for the second year, $150; for any subsequent year, $50. Students may enter under either the old or the new plan, at their option, in September, 1875, but no degree will be given under the old plan after February, 1877.

For further information address

**T. H. CHANDLER, Dean,**
**222 Tremont Street, Boston, Mass.**

Jan-5

### HARVARD UNIVERSITY.
### Dental Department.

BOSTON, MASS. 1875–76.

### FACULTY.

CHARLES WILLIAM ELIOT, L.L.D., *President.*
OLIVER W. HOLMES, M.D., Professor of Anatomy.
HENRY J. BIGELOW, M.D., Professor of Surgery and Clinical Surgery.
THOMAS H. CHANDLER, D.M.D., Professor of Mechanical Dentistry.
GEORGE T. MOFFATT, M.D., D.M.D., Professor of Operative Dentistry.
LUTHER D. SHEPARD, D.D.S., Adjunct Professor of Operative Dentistry.
NATHANIEL W. HAWES, Assistant Professor of Operative Dentistry.
EDWARD S. WOOD, M.D., Assistant Professor of Chemistry.
HENRY P. BOWDITCH, M.D., Assistant Professor of Physiology.
WILLIAM H. ROLLINS, D.M.D., Instructor in Dental Pathology.
CHARLES A. BRACKETT, D.M.D., Instructor in Dental Therapeutics.
IRA A. SALMON, D.D.S., University Lecturer on Operative Dentistry.
CHARLES B. PORTER, M.D., Demonstrator of Practical Anatomy.
CHARLES WILSON, D.M.D., Demonstrator in Charge.
GEORGE F. GRANT, D.M.D., Demonstrator of Mechanical Dentistry.

The Winter Session of the Eighth year of this School begins on the 30th of September and continues nineteen weeks. The Spring Session begins on Feb. 16th and ends June 29th. Two full Winter Courses are required for graduation. The spring session is simply to take the place of private pupilage, designed chiefly for those who wish to spend their whole three years of study in the school. Unsurpassed facilities for operative and mechanical practice are furnished by the infirmary of the Massachusetts General Hospital which is open throughout the year.

☞ The University Degree, D.M.D. (*Dentariæ Medicinæ Doctor*), is conferred upon those who fulfill the requirements.

### FEES.

**Matriculation, $5.00. Spring Session, $50.00. Winter Session, $110.00.**
**For the Year, $150.00. Graduation, $30.00.**

From and after September 28, 1876, an entire change will be made in the scheme of Instruction. Instruction will be given by lectures, recitations, clinical teaching and practical exercises, uniformly distributed throughout the academic year, and the distinction of winter and summer sessions will thereafter be abolished.

The course of instruction will be progressive, extending over two years.

The regular examinations will be held in the following order, viz.: At the end of the first year, anatomy, including dissection, physiology and general chemistry. A certificate from the demonstrator of anatomy will be required of each student, that he has satisfactorily dissected the three parts of the body.

At the end of the second year, dental pathology, including a knowledge of gestation and diseases of woman so far as they affect the mouth and throat, dental materia medica and therapeutics, oral surgery and surgical pathology, operative and mechanical dentistry. The examinations in operative and mechanical dentistry will include actual operations and the preparation of specimens of mechanical dentistry.

Every candidate must be twenty-one years of age, and of good moral character; he must give evidence of having studied medicine or dentistry three full years; he must have spent at least one continuous year at this school, have presented a satisfactory thesis and have passed all the required examinations.

### FEES UNDER THE NEW PLAN.

There shall be no fees for matriculation, for the diploma, nor for the demonstrators. For the first year a student is a member of the school, the fee shall be $200; for the second year, $150; for any subsequent year, $50. Students may enter under either the old or the new plan, at their option, in September, 1875, but no degree will be given under the old plan after February, 1877.

For further information address

**T. H. CHANDLER, Dean,**
**222 Tremont Street, Boston, Mass.**

### HARVARD UNIVERSITY.

### Dental Department.

### BOSTON, MASS. 1875–76.

### FACULTY.

CHARLES WILLIAM ELIOT, L.L.D., *President.*
OLIVER W. HOLMES, M.D., Professor of Anatomy.
HENRY J. BIGELOW, M.D., Professor of Surgery and Clinical Surgery.
THOMAS H. CHANDLER, D.M.D., Professor of Mechanical Dentistry.
GEORGE T. MOFFATT, M.D., D.M.D., Professor of Operative Dentistry.
LUTHER D. SHEPARD, D.D.S., Adjunct Professor of Operative Dentistry.
NATHANIEL W. HAWES, Assistant Professor of Operative Dentistry.
EDWARD S. WOOD, M.D., Assistant Professor of Chemistry.
HENRY P. BOWDITCH, M.D , Assistant Professor of Physiology.
WILLIAM H. ROLLINS, D M.D., Instructor in Dental Pathology.
CHARLES A. BRACKETT, D.M.D., Instructor in Dental Therapeutics.
IRA A. SALMON, D.D.S., University Lecturer on Operative Dentistry.
CHARLES B. PORTER, M.D., Demonstrator of Practical Anatomy.
CHARLES WILSON, D.M.D., Demonstrator in Charge.
GEORGE F. GRANT, D.M.D., Demonstrator of Mechanical Dentistry.

The Winter Session of the Eighth year of this School begins on the 30th of September and continues nineteen weeks. The Spring Session begins on Feb. 16th and ends June 29th. Two full Winter Courses are required for graduation. The spring session is simply to take the place of private pupilage, designed chiefly for those who wish to spend their whole three years of study in the school. Unsurpassed facilities for operative and mechanical practice are furnished by the infirmary of the Massachusetts General Hospital which is open throughout the year.

☞ The University Degree, D.M.D. (*Dentariæ Medicinæ Doctor*), is conferred upon those who fulfill the requirements.

### FEES.

**Matriculation, $5.00. Spring Session, $30.00. Winter Session, $110.00.**
**For the Year, $150.00. Graduation, $30.00.**

From and after September 28, 1876, an entire change will be made in the scheme of Instruction. Instruction will be given by lectures, recitations, clinical teaching and practical exercises, uniformly distributed throughout the academic year, and the distinction of winter and summer sessions will thereafter be abolished.

The course of instruction will be progressive, extending over two years.

The regular examinations will be held in the following order, viz.: At the end of the first year, anatomy, including dissection, physiology and general chemistry. A certificate from the demonstrator of anatomy will be required of each student, that he has satisfactorily dissected the three parts of the body.

At the end of the second year, dental pathology, including a knowledge of gestation and diseases of woman so far as they affect the mouth and throat, dental materia medica and therapeutics, oral surgery and surgical pathology, operative and mechanical dentistry. The examinations in operative and mechanical dentistry will include actual operations and the preparation of specimens of mechanical dentistry.

Every candidate must be twenty-one years of age, and of good moral character; he must give evidence of having studied medicine or dentistry three full years; he must have spent at least one continuous year at this school, have presented a satisfactory thesis and have passed all the required examinations.

### FEES UNDER THE NEW PLAN.

There shall be no fees for matriculation, for the diploma, nor for the demonstrators. For the first year a student is a member of the school, the fee shall be $200; for the second year, $150; for any subsequent year, $50. Students may enter under either the old or the new plan, at their option, in September, 1875, but no degree will be given under the old plan after February, 1877.

For further information address

**T. H. CHANDLER, Dean,**

**222 Tremont Street, Boston, Mass.**

### PENNSYLVANIA COLLEGE OF DENTAL SURGERY.

*The Nineteenth Annual Session, 1875—'76.*

### FACULTY.

T. L. BUCKINGHAM, D. D. S., Professor of Chemistry.
. WILDMAN, M. D., D. D. S., Professor of Mechanical Dentistry and Metallurgy.
G. T. BARKER, D. D. S., Professor of Dental Pathology and Therpuetics.
JAMES TRUMAN, D. D. S., Professor of Dental Histology and Operative Dentistry.
JAS. TYSON, M. D., Professor of Physiology and Microscopic Anatomy.
J. EWING MEARS. M. D., Professor of Anatomy and Surgery.
CHARLES W. MELONEY, D. D. S., Demonstrator of Operative Dentistry.
A. B. ABEL, Jr., D. D. S., Demonstrator of Mechanical Dentistry.
A. H. HENDERSON, D. D. S., Assistant Demonstrator of Operative Dentistry.
H. Y. EASTLACK, D. D. S., Assistant Demonstrator of Mechanical Dentistry.

### CLINICAL INSTRUCTORS.

A. LAWRENCE, M. D., D. D. S.

A. L. NORTHROP, D. D. S.

C. A. MARVIN, D. D. S.

C. PALMER, D. D. S.

E. T. DARBY, D. D. S.

R. HUEY, D. D. S.

W. H. TRUEMAN, D. D. S.

The Infirmary and Laboratory will be opened on the fifteenth of March, and remain open during the entire sessions, when ample opportunities will be afforded the student for the prosecution of the practical part of his studies under the guidance and supervision of demonstrators of known integrity and capability. During the Fall Course and the Regular or Winter Session, a Clinical Lecture will be given and Operations performed by one of the Professors every Saturday.

### THE SPRING AND FALL SESSIONS.

THE SPRING COURSE of Lectures will commence on the first Monday in April and continue until the last of June. Fee for the course, fifty dollars.

THE FALL COURSE will commence on the second Monday in September, and continue until the last of October, and will be free to those who Martriculate for the regular session.

During the Spring and Fall Sessions there will be one Lecture a day, from 5 to 6 o'clock, P. M., and, with the exception of Saturday, the Dispensary and Laboratory will be open daily for six hours.

The attendance of the entire Spring and Fall courses will be deemed equivalent to the term of pupilage under a private preceptor during the recess of the regular sessions.

### THE REGULAR SESSION.

Will commence on the first Monday in November, and continue until the first of March ensuing. The course is so arranged that eighteen lectures will be delivered each week on the various branches taught in the College.

### FEES.

| | |
|---|---|
| Martriculation (paid but once) | $5.00 |
| For the Course (Demonstrators' ticket included) | 100.00 |
| Diploma | 30.00 |

For further information, address

E. WILDMAN, Dean,

**1205 Arch St., Philadelphia.**

Board from 5 to $8 per week. All instruments requirad can be procured for 20 to $25.

### HARVARD UNIVERSITY.

### Dental Department.

### BOSTON, MASS. 1875–76.

**FACULTY.**

CHARLES WILLIAM ELIOT, L.L.D., *President.*
OLIVER W. HOLMES, M.D., Professor of Anatomy.
HENRY J. BIGELOW, M.D., Professor of Surgery and Clinical Surgery.
THOMAS H. CHANDLER, D.M.D., Professor of Mechanical Dentistry.
GEORGE T. MOFFATT, M.D., D.M.D., Professor of Operative Dentistry.
LUTHER D. SHEPARD, D.D.S., Adjunct Professor of Operative Dentistry.
NATHANIEL W. HAWES, Assistant Professor of Operative Dentistry.
EDWARD S. WOOD, M.D., Assistant Professor of Chemistry.
HENRY P. BOWDITCH, M.D., Assistant Professor of Physiology.
WILLIAM H. ROLLINS, D.M.D., Instructor in Dental Pathology.
CHARLES A. BRACKETT, D.M.D., Instructor in Dental Therapeutics.
IRA A. SALMON, D.D.S., University Lecturer on Operative Dentistry.
CHARLES B. PORTER, M.D., Demonstrator of Practical Anatomy.
CHARLES WILSON, D.M.D., Demonstrator in Charge.
GEORGE F. GRANT, D.M.D., Demonstrator of Mechanical Dentistry.

The Winter Session of the Eighth year of this School begins on the 30th of September and continues nineteen weeks. The Spring Session begins on Feb. 16th and ends June 29th. Two full Winter Courses are required for graduation. The spring session is simply to take the place of private pupilage, designed chiefly for those who wish to spend their whole three years of study in the school. Unsurpassed facilities for operative and mechanical practice are furnished by the infirmary of the Massachusetts General Hospital which is open throughout the year.

☞ The University Degree, D.M.D. (*Dentariæ Medicinæ Doctor*), is conferred upon those who fulfill the requirements.

**FEES.**

**Matriculation, $5.00. Spring Session, $50.00. Winter Session, $110.00.**
**For the Year, $150.00. Graduation, $30.00.**

From and after September 28, 1876, an entire change will be made in the scheme of Instruction. Instruction will be given by lectures, recitations, clinical teaching and practical exercises, uniformly distributed throughout the academic year, and the distinction of winter and summer sessions will thereafter be abolished.

The course of instruction will be progressive, extending over two years.

The regular examinations will be held in the following order, viz.: At the end of the first year, anatomy, including dissection, physiology and general chemistry. A certificate from the demonstrator of anatomy will be required of each student, that he has satisfactorily dissected the three parts of the body.

At the end of the second year, dental pathology, including a knowledge of gestation and diseases of woman so far as they affect the mouth and throat, dental materia medica and therapeutics, oral surgery and surgical pathology, operative and mechanical dentistry. The examinations in operative and mechanical dentistry will include actual operations and the preparation of specimens of mechanical dentistry.

Every candidate must be twenty-one years of age, and of good moral character; he must give evidence of having studied medicine or dentistry three full years; he must have spent at least one continuous year at this school, have presented a satisfactory thesis and have passed all the required examinations.

**FEES UNDER THE NEW PLAN.**

There shall be no fees for matriculation, for the diploma, nor for the demonstrators. For the first year a student is a member of the school, the fee shall be $200; for the second year, $150; for any subsequent year, $50. Students may enter under either the old or the new plan, at their option, in September, 1875, but no degree will be given under the old plan after February, 1877.

For further information address

**T. H. CHANDLER, Dean,**

**222 Tremont Street, Boston, Mass.**

### Ohio College of Dental Surgery.

The Thirtieth Annual Session of the Ohio College of Dental Surgery will begin on the 13th of October, 1875, and continue till March following.

### FACULTY.

JAMES TAYLOR, M. D., D. D. S., Emeritus Institutes of Dental Science.
WM. CLENDENIN, M. D., Anatomy.
J. L. CILLEY, M. D., Physiology and Histology.
F. BRUNNING, M. D., Pathology and Therapeutics.
J. TAFT, D. D. S., Operative Dentistry, Hygiene, Microscopy.
J. S. CASSIDY, D. D. S., Chemistry.
J. I. TAYLOR, D. D. S., H. M. REID, D. D. S., Clinical Dentistry.
WM. VAN ANTWERP, D. D. S., Mechanical Dentistry.
J. L. CILLEY, M. D., Practical Anatomy.

| | |
|---|---|
| Matriculation Fee (but once) | $ 5 00 |
| Professors' Tickets for one session | 100 00 |
| Or for Winter and Spring terms | 130 00 |
| Demonstrator's Ticket (for Anatomy) | 5 00 |
| Diploma Fee | 30 00 |

### TEXT BOOKS.

Gray's or Wilson's Anatomy; Dalton's, Carpenter's or Kirke's Physiology; U. S. Dispensatory; Fowne's, Turner's or Graham's Chemistry; Watt's Chemical Essays; Taft's Operative Dentistry; Richardson's Mechanical Dentistry; Harris' Principles and Practice of Dental Science: Tome's Dental Surgery; Harris' Dictionary of Dental Surgery.

### TERMS OF GRADUATION.

A candidate for graduation must have two full years of pupilage, part of which, at least, should be with a reputable dental practitioner, and good teacher, inclusive of two complete courses of lectures in a Dental College.

A graduate of a respectable Medical College, who has had one year's pupilage under a reputable Dentist, or a student having had a regular pupilage of two years and four years practice, and otherwise satisfactory may be admitted to the Senior Class, and to examination for the degree of D. D. S., after attending one full course of lectures.

For further particulars address the Dean or Secretary by letter.

Students coming to the city should call on the Dean. Good board can be had for $4 00 to $7 00 a week.

### SPRING COURSE, 1876.

The Spring course in this Institution will commence on the 15th of March, and continue till the 1st of June.

Tickets for the course, $50, to be paid on entering, or $130 for Spring and Winter term.

J. TAFT. DEAN,

117 West 4th St.

F. BRUNNING, SEC.

### HARVARD UNIVERSITY.
### Dental Department.
### BOSTON, MASS. 1875–76.

### FACULTY.

Charles William Eliot, L.L.D., *President.*
Oliver W. Holmes, M.D., Professor of Anatomy.
Henry J. Bigelow, M.D., Professor of Surgery and Clinical Surgery.
Thomas H. Chandler, D.M.D., Professor of Mechanical Dentistry.
George T. Moffatt, M.D., D.M.D., Professor of Operative Dentistry.
Luther D. Shepard, D.D.S., Adjunct Professor of Operative Dentistry.
Nathaniel W. Hawes, Assistant Professor of Operative Dentistry.
Edward S. Wood, M.D., Assistant Professor of Chemistry.
Henry P. Bowditch, M.D., Assistant Professor of Physiology.
William H. Rollins, D.M.D., Instructor in Dental Pathology.
Charles A. Brackett, D.M.D., Instructor in Dental Therapeutics.
Ira A. Salmon, D.D.S., University Lecturer on Operative Dentistry.
Charles B. Porter, M.D., Demonstrator of Practical Anatomy.
Charles Wilson, D.M.D., Demonstrator in Charge.
George F. Grant, D.M.D., Demonstrator of Mechanical Dentistry.

The Winter Session of the Eighth year of this School begins on the 30th of September and continues nineteen weeks. The Spring Session begins on Feb. 16th and ends June 29th. Two full Winter Courses are required for graduation. The spring session is simply to take the place of private pupilage, designed chiefly for those who wish to spend their whole three years of study in the school. Unsurpassed facilities for operative and mechanical practice are furnished by the infirmary of the Massachusetts General Hospital which is open throughout the year.

☞ The University Degree, D.M.D. (*Dentariæ Medicinæ Doctor*), is conferred upon those who fulfill the requirements.

### FEES.

**Matriculation, $5.00. Spring Session, $50.00. Winter Session, $110.00.**
**For the Year, $150.00. Graduation, $30.00.**

From and after September 28, 1876, an entire change will be made in the scheme of Instruction. Instruction will be given by lectures, recitations, clinical teaching and practical exercises, uniformly distributed throughout the academic year, and the distinction of winter and summer sessions will thereafter be abolished.

The course of instruction will be progressive, extending over two years.

The regular examinations will be held in the following order, viz.: At the end of the first year, anatomy, including dissection, physiology and general chemistry. A certificate from the demonstrator of anatomy will be required of each student, that he has satisfactorily dissected the three parts of the body.

At the end of the second year, dental pathology, including a knowledge of gestation and diseases of woman so far as they affect the mouth and throat, dental materia medica and therapeutics, oral surgery and surgical pathology, operative and mechanical dentistry. The examinations in operative and mechanical dentistry will include actual operations and the preparation of specimens of mechanical dentistry.

Every candidate must be twenty-one years of age, and of good moral character; he must give evidence of having studied medicine or dentistry three full years; he must have spent at least one continuous year at this school, have presented a satisfactory thesis and have passed all the required examinations.

### FEES UNDER THE NEW PLAN.

There shall be no fees for matriculation, for the diploma, nor for the demonstrators. For the first year a student is a member of the school, the fee shall be $200; for the second year, $150; for any subsequent year, $50. Students may enter under either the old or the new plan, at their option, in September, 1875, but no degree will be given under the old plan after February, 1877.

For further information address

**T. H. CHANDLER, Dean,**
**222 Tremont Street, Boston, Mass.**

AMERICAN ACADEMY OF DENTAL SURGERY met in New York April 7, 1875. *Pres.* Geo. H. Perrine; *Rec. Sec.* Geo. A. Wilson; *Cor. Sec.* J. A. Bishop.

AMERICAN DENTAL ASSOCIATION—Meets on the first Tuesday in Aug., 1870, at Philadelphia. *Pres.* A. L. Northrup, New York; *Cor. Sec.* J. N. McQuilien, Philadel'a; *Rec. Sec.* C. Stodard Smith, Springfield, Ill.

*American Dental Convention* Meets at Philadelphia, first Tuesday in August, 1876. *Pres.* B. F. Coy, Baltimore: *Cor. Sec.* D. Roberts, Philadelphia, Pa.; *Rec. Sec.* A. Tees, Philadelphia, Pa.

*List of Societies Represented in the American Dental Association.*

*American Academy of Dental Science* Meets at Boston, Mass. *Pres.* Joshua Tucker M. D., of Boston ; *Sec.* W. L. Tucker, of Boston ; *Cor. Sec.*, E. N. Harris.

*Brooklyn Dental Society* Meets semi-monthly. *Pres.* M. E. Elmandorf; *Rec. Sec.* C. P. Crandell ; *Cor. Sec.* A. N. Chapman.

*Buffalo Dental Society* Meets second Monday of each month at Buffalo. *Pres.* S. A. FREEMAN ; *Rec. Sec.* G. C. DABOLL.

*Boston Dental Institute*—Meets first Tuesday of each month. *Pres.* D. G. WILLIAMS, Buffalo; *Cor. Sec.* D. S. Dickerman.

*California State Dental Association* Meets annually in June. *Pres.* C. C. Knowles, San Francisco ; *Rec. Sec.* H. J. Plomteaux, Woodland.

*Chicago Dental Society* Meets monthly at Chicago. *Pres.* C. R. E. Koch; *Rec. Sec.* D. B. Freeman.

*Connecticut Valley Dental Association.* Meets semi-annually, second Tuesday of June and October. *Pres.* J. J. Anderson; *Rec. Sec.* L. C. Taylor, Holyoke, Mass. Next Meeting in Northampton, June, 1875

*Connecticut State Dental Society.* *Pres.* R. C. Dunham, New Britain ; *Rec. Sec.*, D. E. Lane, Hartford.

*Charleston Dental Association*—Meets second Tuesday of every month ; *Pres.* T. F. Chupein ; *Sec. and Treas.* B. A. Muckenfuss.

*Delaware Dental Association*—Meets semi-annually. *Pres.* E. W. Haines, Newark ; *Sec.* C. R. Jeffries, Wilmington.

*Eastern Indiana Dental Association.*—Meets semi-annually, first Tuesday in May and last Tuesday in October; *Pres.* J. K. Jameson, Connersville; *Sec.* J. W. White, Knightstown, Ind. (Place of meeting fixed by appointment.)

*Eastern Ohio Dental Association*—Meets on Tuesday, August 26th, 1873, at Alliance ; *Pres.* A. J. Douds ; *Sec.* J. . Porter.

*East Tennessee Dental Association*—Meets annually at Knoxville, Tenn. *Pres.* Wm. W. F. Fowler; *Rec. Sec.* S. M. Prothro.

*Forest City Society of Dental Surgeons*—*Pres.* B. Strickland, Cleveland, *Rec. Sec.* H. L. Ambler, Cleveland ; *Cor. Sec.* Chas. Buffett, Cleveland.

*Georgia State Dental Society* Next meeting at Atlanta, 2d Tuesday in May, 1876. *Pres.* G. W. McEllaney; *Cor. Sec.* C. C. Allen, Marietta.

*Harris Dental Association* Meets quarterly on the first Thursday in February, May. August, November. *Pres.* P. G. Austand, Millersville; *Sec.* W. N. Amer, Lancaster,

*Hudson River Association of Dental Surgeons* Meets on the second Wednesdays of May, September and January. *Pres.* L. S. Straw, Newburg, N. Y ; *Rec. Sec.* T. W. DuBois, Poughkeepsie, N. Y.

*Hudson Valley Dental Association* Meets monthly at Troy, N. Y. *Pres.* L. C. Wheeler, *Rec. Sec.* S. G. Andres ; *Cor. Sec.* S. D. French.

*Illinois State Dental Soeiety* Meets annually. *Pres.* E. D. Swain, Chicago; *Sec.* R. E. Coch; Chicago. Meets second Tuesday in May, 1876, at Galesburg.

*Indiana State Dental Society*—Meets next year at Indianapolis, on the first Tuesday of June. *Pres.* J. B. Clayton, Shelbyville; *Cor. and Rec. Sec.* P. McDonald, Goshen.

*Iowa State Dental Society*—Meets annually. Next meeting at Burlington, on the second Tuesday of May. *Pres.* C. P. Artman Waterloo; *Rec. Sec.* R. L. Cochran Burlington; *Cor. Sec.* I. P. Wilson, Burlington.

*Kansas State Dental Society* Meets annually. Next meeting at Leavanworth, first Wednesday in May, 1875. *Pres.* L. C. Wasson; *Sec.* J. D Patterson.

*Kansas and Missouri Valley Dental Association*—Meets first and third Tuesdays of erch month. *Pres.* J. R. Stark ; *Sec.* H. C. Massie, Kansas City, Mo.

*Kentucky State Dental Association* Meets annually first Tuesday in June, next meeting in Louisville. *Pres.*, C. E Dunn; *Sec.*, A. C. Rawls.

*Lake Erie Dental Association* Meets Annually. *Pres.* C. B. Ansart, Oil City, Pa. *Sec.* C. D. Elliot, Franklin, Pa. Next meeting first Tuesday of May, 1874.

*Mad River Dental Association* Meets at Dayton on the first Tuesdays in April and October. *Pres.* S. B. Tizzard, Dayton ; *Sec.* A. J. Grosvenor, Troy.

*Maine Dental Society*—Meets in Aug., at Thomaston. *Pres.* E. J. Roberts, Augusta ; *Sec.* C. E. Hussey, Biddeford.

*Maryland Association of Dentists* Meets the last Thursday of every month. *Pres.* J. B. Bean ; *Sec.* S. M. Field.

*Massachusetts Dental Society*—Meets monthly. *Pres.* J. H. Batchelder, of Salem *Sec.* C. Wilson ; *Cor. Sec.* L. D. Shepard.

*Michigan State Dental Association*—Meets annually second Tuesday of Oct., at Grand Rapids. *Pres.* T. R. Perry, Grand Rapids; *Rec. & Cor. Sec.* Wm. Tremper, Ypsilanti

*Mississippi Valley Dental Association* Meets annually at Cincinnati, on first Wednesday in March, 1876. *Pres.* George Watt, Xenia, Ohio; *Rec. Sec.* Wm. Taft, Cin.
*Mississippi State Dental Association* will meet 3d Wednesday of August. *Pres.* J. D. Miles; *Sec.* A. Riser.
*Minnesota Dental Associrtion* Meets annually. Next meeting at Minneapolis; May, 1876. *Pres.* F. A. Williamson, Reading, *Sec.* W. F. Lewis, Winona-St. Paul.
*Missouri Dental Association* Meets on the first Tuesday of June, in St. Louis. *Pres.* C. W. Rivers, St. Louis; *Sec.* W. H. Evans, St. Louis.
*Missouri Valley Dental Association** Meets on the fourth Tuesday in July, in Nebraska City. *Pres.* A. S. Billings, Omaha, Neb.; *Sec. and Treas.* J. F. Sanborn, Tabor, Iowa.
*Merrimac Valley Dental Association* Meets semi-annually first Tuesday of Oct. and May.* *Pres.* L. H. Kidder, Mass; *Rec. Sec.* A. M. Dudley, of Lowell; *Cor. Sec.* D. T. Porter, Lawrence, Mass.
*Memphis Dental Society*—Meets on the first Tuesday of each month. *Pres.* W. M. Arrington; *Sec.* V. F. Elliott; *Treas.* C. L. Harris.

*Nashville Dental ssociation*—Meets on the second Tuesday of each month. *Pres.* J. C. Ross; *Sec.* R. R. Freeman; *Cor. Sec.* S. J. Cobb.
*New Jersey State Dental Society*—Meets annually. Next meeting at Long Branch, on the first Tuesday in July, 1875. *Pres.* Geo. C. Brown, Mount Holly; *Sec.* J. W. Scarborough, Lambertville.
*New Haven Dental Society*—Meets on the first Wednesday of each month at New Haven. *Pres.* J. T. Metcalf, *Rec and Cor. Sec.* C. L. Smith, of New Haven.
*New Orleans Dental Society* Meets monthly. *Pres.* ———; *Rec. Sec.* A. F. McLane.
*Northern Ohio Dental Association.* Meets annually, second Tuesday of June, 1876, at Cleveland. *Pres.* E. J. Way, Sandusky; *Cor. Sec.* C. Buffett, Clinton.
*Northern Iowa Dental Association*—Meets annually, on the second Tuesday in June, 1873. Next meeting at Waterloo; *Pres.* J. N. Abbott, Lancaster, *Rec. Sec.* A. V Eaton, Anamosa; *Cor. Sec.* A. R. Mason, Waterloo.

*Ohio College Dental Association*—Meets annually Feb. 25. at Cincinnati. *Pres.* Jas. Leslie, Cin'ti.; *Rec. Sec.* H. A. Smith, of Cincinnati.
*Ohio State Dental Association*—Meets annually at Columbus. Next meeting, first Tuesday of December. *Pres.* C. R. Butler; *Rec. Sec.* F. A. Hunter, Cincinnati; *Cor. Sec.* C. R. Butler, Cleveland, O.
*Odontographic Society of Pennsylvania*—Meets monthly in Philadelphia. Next meeting, Wednesday, Sept. 1st. *Pres.* Louis Jack; *Rec. Sec.* E. L. Hewitt.
*Odontological Society of New York City* Meets the second Tuesday of each month. *Pres.* A. L. Northrup; *Rec. Sec.* Wm. Jarvie, Jr.
*Old Colony Dental Association* Meets quarterly.* *Pres.* C. G. Davis; *Rec. Sec.* L. W. Puffer, North Bridgewater, Mass; *Cor. Sec.* N. C. Fowler, Yarmouth, Mass.
*Ohio Valley Dental Association* Meets annually, in N. E. Ky; this year in Paris, Ky. Tuesday, Sept. 9th.
*Oregon State Dental Society* Meets annually. *Pres.* J. H. Hatch, Portland; *Rec. Sec.* J. R. Cardwell, Portland.

*Penn. Central Dental Asso.* meets on the 1st Tuesday of Nov. 1875, at Allentown. *Pres.* F. Hickman, *Sec.* R. E. Diefenderfer.
*Pennsylvania State Dental Society* meets next at Cresson Springs 2d Tues. of July, 1875 *Pres.* J. H. McQuillen; *Cor. Sec.* M. H. Webb.
*Poughkeepsie Dental Society* Meets monthly. *Pres.* J. H. Mann; *Sec.* H. F. Clark
*Pennsylvania Association of Dental Surgery* Meets monthly in Philadelphia. *Pres* Spencer Roberts; *Sec.* E. R. Pettit.

*San Francisco Dental Association* Meets monthly. *Pres.* C. C. Knowles; *Cor. Se* H. E. Knox; *Rec. Sec.* Wm. Dutch.
*St. Louis Dental Society* Meets monthly. *Pres.* W. H. Morrison; *Sec.* A. H. Fuller.
*Susquehanna Dental Association* Meets annually.* *Pres.* C. S. Beck: *Rec. Sec.* W H. Nertz. Meets May, 1875, at Scranton.
*Society of Dental Surgeons of the City of New York* Meets semi-monthly in New York. *Pres.* B. F. Franklin: *Rec. Sec.* J. S. Latimer; *Cor. Sec.* E. H. Burras.
**Southern Dental Association* Meets annually. Next meeting at Memphis, Tenn., at time of Mardi-Gras. *Pres.* J. R. Walker, New Orleans, La.; *Rec. Sec.* J. F Thompson Va
*South Carolina State Dental Association* Meets annually. *Pres.* G. F. S. Wright, Columbia; *Sec.* F. T. Chupein, Charleston; *Cor. Sec.* J. S. Thompson, Abbeville. Next meeting at Greenville, S. C., second Thursday of June, 1876.

*Tennessee Dental Association* Meets semi-annually. Next meeting in Nashville, on the 4th Wednesday in June. 1876. *Pres.* R. R. Freema; *Rec. Sec.* H. W. Morgan, *Cor. Sec.* L. G. Noel.
*Virginia State Dental Association* Meets in Richmond, first Thursday in Nov. of each year. *Pres.* H. M. Grant, Abington: *Sec.* G. F. Kersee, Richmond.

*Washington, City Dental Society* Meets annually. *Pres.* R. F. Hunt, *Sec.* Dr. Evans
*Wabash Valley Dental Association* Meets annually. Next meeting at Wayne, third Tuesday in March, 1872. *Pres.* A. B. Cunningham, Attica: *Sec.* S. B. Brown, Fort Wayne.
* *Wisconsin State Dental Association* Meets semi-annually. Next meeting at Watertown. second Tuesday of January, 1872, *Pres.* Edgar Palmer, Le Crook *Sec.* C. C. Chittenden, Madison.

### State and District Dental Societies of State of New York.

*New York State Dental Society*--Meets at Albany on the second Wednesday of July next. *Pres.* W. C. Barrett, Warsaw, N. Y.; *Cor. Sec.* S. B. Palmer; *Rec. Sec.* S. S. A, Freeman, Buffalo.

*First District* Meets second and fourth Wednesday evenings of each month. Annual meeting in New York, first Tuesday in June. *Pres.* J. S. Latiner New York; *Rec. Sec.* F. M. Odell, New York.

*Second District.* Annual meeting in Brooklyn, first Monday in April. *Pres.* W. S. Elliott; *Rec. Sec.* M. E. Elmandorf. Brooklyn; *Cor. Sec.* F. W. Dalbare.

*Third District.* Meets semi-annually on second Tuesday in January and July. Annual meeting at Albany on second Tuesday in January. *Pres.* S. P. Welsh; *Rec. Sec.* H. A. Hall, Troy; *Cor. Sec.* J. A. Perkins, Albany.

*Fourth District.* Meets semi-annually. Meets annually at Saratoga on second Tuesday in June. *Pres.* C, A. Elmore, Ft. Edward; *Rec. Sec.* E. S. Pearsall, Saratoga.

*Fifth District.* Meets semi-annually. Annual meeting at Syracuse. second Tuesday in June. *Pres.* J. D. Huntington, Watertown; *Rec. Sec.* F. D. Nellis, Syracuse; *Cor. Sec.* J. S. Marshall, Syracuse.

*Sixth District.* Meets semi-annually. Annual meeting at Binghampton on second Tuesday in June. *Pres.* Frank B. Darby, Oswego; *Rec. Sec.* F. E. Perry, Norwich; *Cor. Sec.* A. M. Holmes, Morrisville.

*Seventh District.* Meets semi-annually. Annual meeting at Rochester first Tuesday in June. *Pres.* H. S. Miller; *Rec. Sec.* J. E. Line, Rochester.

*Eighth District.* Meets semi-annually. Annual on second Tuesday in May. *Pres* G. C. Daboll, *Rec. Sec.* S. A. Freeman, *Cor. Sec.* T. G. Lewis, Buffalo.

*The Seventh and Eighth District Societies* hold a union meeting on the last Tuesday of October. alternately at Buffalo and Rochester.

The Ohio State Board of Dental Examiners will meet in Columbus on the first Tuesday of Dec. 1875, at 10 o'clock. *Pres.* J. Taft; *Sec.* W. P. Horton.

### Exchanges.

ST. LOUIS MEDICAL AND SURGICAL JOURNAL.
BOSTON MEDICAL AND SURGICAL JOURNAL.
THE PACIFIC MEDICAL AND SURGICAL JOURNAL, SAN FRANCISCO.
PHIDADELPHIA MEDICAL AND SURGICAL JOURNAL.
CINCINNATI MEDICAL ADVANCE.
CINCINNATI LANCET AND OBSERVER.
DENTAL COSMOS, PHILADELPHIA.
LADIES' REPOSITORY, CINCINNATI.
HALL'S JOURNAL OF HEALTH, N. Y.
CHICAGO MEDICAL EXAMINER.
THE DETROIT REVIEW OF MEDICINE AND PHARMACY.
MEDICAL RECORD, N. Y.
NASHVILLE JOURNAL OF MEDICINE AND SURGERY.
AMERICAN JOURNAL OF DENTAL SCIENCE, BALTIMORE.
THE UNITED STATES MEDICAL AND SURGICAL JOURNAL, CHICAGO.
NEW ORLEANS JOURNAL OF MEDICINE.
AMERICAN AGRICULTURIST, N. Y.
BOSTON JOURNAL OF CHEMISTRY.
JOURNAL OF APPLIED CHEMISTRY, N. Y.
CANADA JOURNAL OF DENTAL SCIENCE, MONTREAL.
INDIANA JOURNAL OF MEDICINE.
MEDICAL AND SURGICAL REPORTER, SALEM, OREGON.
MISSOURI DENTAL JOURNAL, ST. LOUIS.
ECLECTIC MEDICAL JOURNAL, CINCINNATI.

### HARVARD UNIVERSITY.

### Dental Department.

### BOSTON, MASS. 1875–76.

---

### FACULTY.

Charles William Eliot, L.L.D., *President.*
Oliver W. Holmes, M.D., Professor of Anatomy.
Henry J. Bigelow, M.D., Professor of Surgery and Clinical Surgery.
Thomas H. Chandler, D.M.D., Professor of Mechanical Dentistry.
George T. Moffatt, M.D., D.M.D., Professor of Operative Dentistry.
Luther D. Shepard, D.D.S., Adjunct Professor of Operative Dentistry.
Nathaniel W. Hawes, Assistant Professor of Operative Dentistry.
Edward S. Wood, M.D., Assistant Professor of Chemistry.
Henry P. Bowditch, M.D., Assistant Professor of Physiology.
William H. Rollins, D.M.D., Instructor in Dental Pathology.
Charles A. Brackett, D.M.D., Instructor in Dental Therapeutics.
Ira A. Salmon, D.D.S., University Lecturer on Operative Dentistry.
Charles B. Porter, M.D., Demonstrator of Practical Anatomy.
Charles Wilson, D.M.D., Demonstrator in Charge.
George F. Grant, D.M.D., Demonstrator of Mechanical Dentistry.

---

The Winter Session of the Eighth year of this School begins on the 30th of September and continues nineteen weeks. The Spring Session begins on Feb. 16th and ends June 29th. Two full Winter Courses are required for graduation. The spring session is simply to take the place of private pupilage, designed chiefly for those who wish to spend their whole three years of study in the school. Unsurpassed facilities for operative and mechanical practice are furnished by the infirmary of the Massachusetts General Hospital which is open throughout the year.

☞ The University Degree, D.M.D. (*Dentariæ Medicinæ Doctor*), is conferred upon those who fulfill the requirements.

---

### FEES.

**Matriculation, $5.00. Spring Session, $50.00. Winter Session, $110.00.**
**For the Year, $150.00. Graduation, $30.00.**

---

From and after September 28, 1876, an entire change will be made in the scheme of Instruction. Instruction will be given by lectures, recitations, clinical teaching and practical exercises, uniformly distributed throughout the academic year, and the distinction of winter and summer sessions will thereafter be abolished.

The course of instruction will be progressive, extending over two years.

The regular examinations will be held in the following order, viz.: At the end of the first year, anatomy, including dissection, physiology and general chemistry. A certificate from the demonstrator of anatomy will be required of each student, that he has satisfactorily dissected the three parts of the body.

At the end of the second year, dental pathology, including a knowledge of gestation and diseases of woman so far as they affect the mouth and throat, dental materia medica and therapeutics, oral surgery and surgical pathology, operative and mechanical dentistry. The examinations in operative and mechanical dentistry will include actual operations and the preparation of specimens of mechanical dentistry.

Every candidate must be twenty-one years of age, and of good moral character; he must give evidence of having studied medicine or dentistry three full years; he must have spent at least one continuous year at this school, have presented a satisfactory thesis and have passed all the required examinations.

### FEES UNDER THE NEW PLAN.

There shall be no fees for matriculation, for the diploma, nor for the demonstrators. For the first year a student is a member of the school, the fee shall be $200; for the second year, $150; for any subsequent year, $50. Students may enter under either the old or the new plan, at their option, in September, 1875, but no degree will be given under the old plan after February, 1877.

For further information address

**T. H. CHANDLER, Dean,**

**222 Tremont Street, Boston, Mass.**

### PENNSYLVANIA COLLEGE OF DENTAL SURGERY,

PHILADELPHIA, PENNA.

*The Twentieth Annual Session, 1875—'76.*

### FACULTY.

T. L. BUCKINGHAM, D. D. S., Professor of Chemistry.

. WILDMAN, M. D., D. D. S., Professor of Mechanical Dentistry and Metallurgy.

G. T. BARKER, D. D. S., Professor of Dental Pathology and Therpuetics.

JAMES TRUMAN, D. D. S., Professor of Dental Histology and Operative Dentistry.

JAS. TYSON, M. D., Professor of Physiology and Microscopic Anatomy.

J. EWING MEARS. M. D., Professor of Anatomy and Surgery.

CHARLES W. MELONEY, D. D. S., Demonstrator of Operative Dentistry.

A. B. ABEL, Jr., D. D. S., Demonstrator of Mechanical Dentistry.
Assistant Demonstrator of Operative Dentistry.

H. Y. EASTLACK, D. D. S., Assistant Demonstrator of Mechanical Dentistry.

### CLINICAL INSTRUCTORS.

A. LAWRENCE, M. D., D. D. S. — C. PALMER, D. D. S.
A. L. NORTHROP, D. D. S. — E. T. DARBY, D. D. S.
C. A. MARVIN, D. D. S. — R. HUEY, D. D. S.
W. H. TRUEMAN, D. D. S.

The Infirmary and Labora*ory will be opened on the fifteenth of March, and remain open during the entire sessions, when ample opportunities will be afforded the student for the prosecution of the practical part of his studies under the guidance and supervision of demonstrators of known integrity and capability. During the Fall Course and the Regular or Winter Session, a Clinical Lecture will be given and Operations performed by one of the Professors every Saturday.

### THE SPRING AND FALL SESSIONS.

THE SPRING OURSE of Lectures will commence on the first Monday in April and continue until the last of June. Fee for the course, fifty dollars.

THE FALL COURSE will commence on the second Monday in September, and continue until the last of October, and will be free to those who Martriculate for the regular session.

During the Spring and Fall Sessions there will be one Lecture a day, from 5 to 6 o'clock, P. M., and, with the exception of Saturday, the Dispensary and Laboratory will be open daily for six hours.

The attendance of the entire Spring and Fall courses will be deemed equivalent to the term of pupilage under a private preceptor during the recess of the regular sessions.

### THE REGULAR SESSION.

Will commence on the first Monday in November, and continue until the first of March ensuing. The course is so arranged that eighteen lectures will be delivered each week on the various branches taught in the College.

### FEES.

| | |
|---|---|
| Martriculation (paid but once) | $5.00 |
| For the Course (Demonstrators' ticket included) | 100.00 |
| Diploma | 30.00 |

For further information, address

E. WILDMAN, Dean,

J.A.S.O. **1205 Arch St., Philadelphia.**

Board from 5 to $8 per week. All instruments requirad can be procured for 20 to $25.

### Ohio College of Dental Surgery.

The Thirtieth Annual Session of the Ohio College of Dental Surgery will begin on the 13th of October, 1875, and continue till March following.

### FACULTY.

JAMES TAYLOR, M. D., D. D. S., Emeritus Institutes of Dental Science.
WM. CLENDENIN, M. D., Anatomy.
J. L. CILLEY, M. D., Physiology and Histology.
F. BRUNNING, M. D., Pathology and Therapeutics.
J. TAFT, D. D. S., Operative Dentistry, Hygiene, Microscopy
J. S. CASSIDY, D. D. S., Chemistry.
J. I. TAYLOR, D. D. S., H. M. REID, D. D. S., Clinical Dentistry.
WM. VAN ANTWERP, D. D. S., Mechanical Dentistry.
J. L. CILLEY, M. D., Practical Anatomy.

| | |
|---|---|
| Matriculation Fee (but once) | $ 5 00 |
| Professors' Tickets for one session | 100 00 |
| Or for Winter and Spring terms | 130 00 |
| Demonstrator's Ticket (for Anatomy) | 5 00 |
| Diploma Fee | 30 00 |

### TEXT BOOKS.

Gray's or Wilson's Anatomy; Dalton's, Carpenter's or Kirke's Physiology; U. S. Dispensatory; Fowne's, Turner's or Graham's Chemistry; Watt's Chemical Essays; Taft's Operative Dentistry; Richardson's Mechanical Dentistry; Harris' Principles and Practice of Dental Science: Tome's Dental Surgery; Harris' Dictionary of Dental Surgery.

### TERMS OF GRADUATION.

A candidate for graduation must have two full years of pupilage, part of which, at least, should be with a reputable dental practitioner, and good teacher, inclusive of two complete courses of lectures in a Dental College.

A graduate of a respectable Medical College, who has had one year's pupilage under a reputable Dentist, or a student having had a regular pupilage of two years and four years practice, and otherwise satisfactory may be admitted to the Senior Class, and to examination for the degree of D. D. S., after attending one full course of lectures.

For further particulars address the Dean or Secretary by letter.

Students coming to the city should call on the Dean. Good board can be had for $4 00 to $7 00 a week.

### SPRING COURSE, 1876.

The Spring course in this Institution will commence on the 15th of March, and continue till the 1st of June. a.s.o.

Tickets for the course, $50, to be paid on entering, or $130 for Spring and Winter term.

J. TAFT. DEAN,

117 West 4th St.

F. BRUNNING, SEC.

### The Dental College of the University of Michigan.

The first annual session of this Institution, will commence on the 1st of October and close on the last Wednesday of March, thus making a course of six months. The regular course of instruction commences at once and will proceed through the term with the customary holiday vacation.

The students in this department, will receive instructions in Amatony, Physiology, Pathology, Chemistry, Materia Medica, Therapeutics and Surgery, from the professor of their respective branches in the *Department* of *Medicine* and Surgery of the university, when lectures commence and continue the same as with dental colleges.

**FACULTY OF THE DENTAL DEPARTMENT.**

J. B. Angell, LL.D,, President.
J, Taft, D.D.S., Professor of Principles and Practice of Operative Dentistry.
J. A. Wattling, D.D.S., Professor of Clinical and Mechanical Dentistry.
W. H. Jackson, Demonstrator.

It is desirable that students should be present at the opening of the session, as the regular course of instruction begins at that time. Seats in the lecture room are assigned by selection to students in the order of registration on the steward's books, and each student is expected to occupy during the session, such seat as he may select. Students on arriving in Ann Arbor, should call at the steward's office.

CONDITION OF GRADUATION.

The candidate must be twenty-one years of age. He must furnish evidence of good moral character.

He must devote three years in the study of his profession, in connection with attendance upon a full course of medical lectures. He must attend two full courses of lectures in the Dental College, or one course elsewhere and the last one here, and we recommend that he attend three courses regularly.

He must sustain an examination satisfactory to the Faculty in all the branches taught.

A graduate of the Medical College may enter the senior class, and if found qualified, may graduate after two years have been devoted to the study of dentistry.

Fees and Expenses.—The fees which must be paid in advance, are as follows:

Martriculation Fff.—Residents of Michigan, $10.00 non residents, $15.00.

Annual Dues.—Residents of Michigan, $15.00, non residents, $20.00.

Gradtation Fff.—For all alike, $5.00. The admission fee is paid but once, and entitles the students to the privileges of permanent membership in any department of the University. The annual tax is paid the first year and every year after·

For further particulars address the Dean of the Dental College, Ann Arbor, Mich.

### PENNSYLVANIA COLLEGE OF DENTAL SURGERY,

PHILADELPHIA, PENNA.

*The Twentieth Annual Session, 1875—'76.*

### FACULTY.

T. L. BUCKINGHAM, D. D. S., Professor of Chemistry.
. WILDMAN, M. D., D. D. S., Professor of Mechanical Dentistry and Metallurgy.
G. T. BARKER, D. D. S., Professor of Dental Pathology and Therpuetics.
JAMES TRUMAN, D. D. S., Professor of Dental Histology and Operative Dentistry.
JAS. TYSON, M. D., Professor of Physiology and Microscopic Anatomy.
J. EWING MEARS, M. D., Professor of Anatomy and Surgery.

CHARLES W. MELONEY, D. D. S., Demonstrator of Operative Dentistry.
A. B. ABEL, Jr., D. D. S., Demonstrator of Mechanical Dentistry.
Assistant Demonstrator of Operative Dentistry.
H. Y. EASTLACK, D. D. S., Assistant Demonstrator of Mechanical Dentistry.

### CLINICAL INSTRUCTORS.

A. LAWRENCE, M. D., D. D. S. — C. PALMER, D. D. S.
A. L. NORTHROP, D. D. S. — E. T. DARBY, D. D. S.
C. A. MARVIN, D. D. S. — R. HUEY, D. D. S.
W. H. TRUEMAN, D. D. S.

The Infirmary and Laboratory will be opened on the fifteenth of March, and remain open during the entire sessions, when ample opportunities will be afforded the student for the prosecution of the practical part of his studies under the guidance and supervision of demonstrators of known integrity and capability During the Fall Course and the Regular or Winter Session, a Clinical Lecture will be given and Operations performed by one of the Professors every Saturday.

### THE SPRING AND FALL SESSIONS.

The Spring Course of Lectures will commence on the first Monday in April and continue until the last of June. Fee for the course, fifty dollars.

The Fall Course will commence on the second Monday in September, and continue until the last of October, and will be free to those who Martriculate for the regular session.

During the Spring and Fall Sessions there will be one Lecture a day, from 5 to 6 o'clock, P. M., and, with the exception of Saturday, the Dispensary and Laboratory will be open daily for six hours.

The attendance of the entire Spring and Fall courses will be deemed equivalent to the term of pupilage under a private preceptor during the recess of the regular sessions.

### THE REGULAR SESSION.

Will commence on the first Monday in November, and continue until the first of March ensuing. The course is so arranged that eighteen lectures will be delivered each week on the various branches taught in the College.

### FEES.

| | |
|---|---|
| Martriculation (paid but once) | $5.00 |
| For the Course (Demonstrators' ticket included) | 100.00 |
| Diploma | 30.00 |

For further information, address

E. WILDMAN, Dean,
**1205 Arch St., Philadelphia.**

J.A.S.O.

Board from 5 to $8 per week. All instruments requirad can be procured for 20 to $25.

5

### Ohio College of Dental Surgery.

The Thirtieth Annual Session of the Ohio College of Dental Surgery will begin on the 13th of October, 1875, and continue till March following.

### FACULTY.

James Taylor, M. D., D. D. S., Emeritus Institutes of Dental Science.
Wm. Clendenin, M. D., Anatomy.
J. L. Cilley, M. D., Physiology and Histology.
F. Brunning, M. D., Pathology and Therapeutics.
J. Taft, D. D. S., Operative Dentistry, Hygiene, Microscopy
J. S. Cassidy, D. D. S., Chemistry.
J. I. Taylor, D. D. S., H. M. Reid, D. D. S., Clinical Dentistry.
Wm. Van Antwerp, D. D. S., Mechanical Dentistry.
J. L. Cilley, M. D., Practical Anatomy.

| | |
|---|---|
| Matriculation Fee (but once) | $ 5 00 |
| Professors' Tickets for one session | 100 00 |
| Or for Winter and Spring terms | 130 00 |
| Demonstrator's Ticket (for Anatomy) | 5 00 |
| Diploma Fee | 30 00 |

### TEXT BOOKS.

Gray's or Wilson's Anatomy; Dalton's, Carpenter's or Kirke's Physiology; U. S. Dispensatory; Fowne's, Turner's or Graham's Chemistry; Watt's Chemical Essays; Taft's Operative Dentistry; Richardson's Mechanical Dentistry; Harris' Principles and Practice of Dental Science: Tome's Dental Surgery; Harris' Dictionary of Dental Surgery.

### TERMS OF GRADUATION.

A candidate for graduation must have two full years of pupilage, part of which, at least, should be with a reputable dental practitioner, and good teacher, inclusive of two complete courses of lectures in a Dental College.

A graduate of a respectable Medical College, who has had one year's pupilage under a reputable Dentist, or a student having had a regular pupilage of two years and four years practice, and otherwise satisfactory may be admitted to the Senior Class, and to examination for the degree of D. D. S., after attending one full course of lectures.

For further particulars address the Dean or Secretary by letter.

Students coming to the city should call on the Dean. Good board can be had for $4 00 to $7 00 a week.

### SPRING COURSE, 1876.

The Spring course in this Institution will commence on the 15th of March, and continue till the 1st of June. a.s.o.

Tickets for the course, $50, to be paid on entering, or $130 for Spring and Winter term.

J. TAFT. Dean,

117 West 4th St.

F. BRUNNING, Sec.

AMERICAN DENTAL ASSOCIATION—Meets on the first Tuesday in Aug., 1870, at Phil delphia. *Pres.* A. L. Northrup, New York; *Cor. Sec.* J. N. McQuilien, Philadel'a; *Rec. Sec.* C. Stodard Smith, Springfield, Ill.

*American Dental Convention* Meets at Philadelphia, first Tuesday in August, 1876. *Pres.* B. F. Coy, Baltimore; *Cor. Sec.* D. Roberts, Philadelphia, Pa.; *Rec. Sec.* A. Tees, Philadelphia, Pa.

*List of Societies Represented in the American Dental Association.*

AMERICAN ACADEMY OF DENTAL SURGERY met in New York April 7, 1875. *Pres.* Geo. H. Perrine; *Rec. Sec.* Geo. A. Wilson; *Cor. Sec.* J. A. Bishop.

*American Academy of Dental Science* Meets at Boston, Mass. *Pres.* Joshua Tucker M. D., of Boston; *Sec.* W. L. Tucker, of Boston; *Cor. Sec.*, E. N. Harris.

*Brooklyn Dental Society* Meets semi-monthly. *Pres.* M. E. Elmandorf; *Rec. Sec.* C. P. Crandell; *Cor. Sec.* A. N. Chapman.

*Buffalo Dental Society* Meets second Monday of each month at Buffalo. *Pres.* S. A. FREEMAN; *Rec. Sec.* G. C. DABOLL.

*Boston Dental Institute*—Meets first Tuesday of each month. *Pres.* D. G. WILLIAMS, Buffalo; *Cor. Sec.* D. S. Dickerman.

*California State Dental Association* Meets annually in June. *Pres.* C. C. Knowles, San Francisco; *Rec. Sec.* H. J. Plomteaux, Woodland.

*Chicago Dental Society* Meets monthly at Chicago. *Pres.* C. R. E. Koch; *Rec. Sec.* D. B. Freeman.

*Connecticut Valley Dental Association.* Meets semi-annually, second Tuesday of June and October. *Pres.* J. J. Anderson; *Rec. Sec.* L. C. Taylor, Holyoke, Mass. Next Meeting in Northampton, June, 1875

*Connecticut State Dental Society.* *Pres.* R. C. Dunham, New Britain; *Rec. Sec.*, D. E. Lane, Hartford.

*Charleston Dental Association*—Meets second Tuesday of every month; *Pres.* T. F. Chupein; *Sec. and Treas.* B. A. Muckenfuss.

*Delaware Dental Association*—Meets semi-annually. *Pres.* E. W. Haines, Newark; *Sec.* C. R. Jeffries, Wilmington.

*Eastern Indiana Dental Association.*—Meets semi-annually, first Tuesday in May and last Tuesday in October; *Pres.* J. K. Jameson, Connersville; *Sec.* J. W. White, Knightstown, Ind. (Place of meeting fixed by appointment.)

*Eastern Ohio Dental Association*—Meets on Tuesday, August 26th, 1873, at Alliance; *Pres.* A. J. Douds; *Sec.* J. . Porter.

*East Tennessee Dental Association*—Meets annually at Knoxville, Tenn. *Pres.* Wm. W. F. Fowler; *Rec. Sec.* S. M. Prothro.

*Forest City Society of Dental Surgeons*—*Pres.* B. Strickland, Cleveland, *Rec. Sec.* H. L. Ambler, Cleveland; *Cor. Sec.* Chas. Buffett, Cleveland.

*Georgia State Dental Society* Next meeting at Atlanta, 2d Tuesday in May, 1876. *Pres.* G. W. McEllaney; *Cor. Sec.* C. C. Allen, Marietta.

*Harris Dental Association* Meets quarterly on the first Thursday in February, May. August, November. *Pres.* P. G. Austand, Millersville; *Sec.* W. N. Amer, Lancaster,

*Hudson River Association of Dental Surgeons* Meets on the second Wednesdays of May, September and January. *Pres.* L. S. Straw, Newburg, N. Y; *Rec. Sec.* T. W. DuBois, Poughkeepsie, N. Y.

*Hudson Valley Dental Association* Meets monthly at Troy, N. Y. *Pres.* L. C. Wheeler, *Rec. Sec.* S. G. Andres; *Cor. Sec.* S. D. French.

*Illinois State Dental Soeiety* Meets annually. *Pres.* E. D. Swain, Chicago; *Sec.* R. E. Coch; Chicago. Meets second Tuesday in May, 1876, at Galesburg.

*Indiana State Dental Society*—Meets next year at Indianapolis, on the first Tuesday of June. *Pres.* J. B. Clayton, Shelbyville; *Cor. and Rec. Sec.* P. McDonald, Goshen.

*Iowa State Dental Society*—Meets annually. Next meeting at Burlington, on the second Tuesday of May. *Pres.* C. P. Artman Waterloo; *Rec. Sec.* R. L. Cochran Burlington; *Cor. Sec.* I. P. Wilson, Burlington.

*Kansas State Dental Society* Meets annually. Next meeting at Leavanworth, first Wednesday in May, 1875. *Pres.* L. C. Wasson; *Sec.* J. D Patterson.

*Kansas and Missouri Valley Dental Association*—Meets first and third Tuesdays of erch month. *Pres.* J. R. Stark; *Sec.* H. C. Massie, Kansas City, Mo.

*Kentucky State Dental Association* Meets annually first Tuesday in June, next meeting in Louisville. *Pres.*, C. E Dunn; *Sec.*, A. C. Rawls.

*Lake Erie Dental Association* Meets Annually. *Pres.* C. B. Ansart, Oil City, Pa. *Sec.* C. D. Elliot, Franklin, Pa. Next meeting first Tuesday of May, 1874.

*Mad River Dental Association* Meets at Dayton on the first Tuesdays in April and October. *Pres.* S. B. Tizzard, Dayton; *Sec.* A. J. Grosvenor, Troy.

*Maine Dental Society*—Meets in Aug., at Thomaston. *Pres.* E. J. Roberts, Augusta; *Sec.* C. E. Hussey, Biddeford.

*Maryland Association of Dentists* Meets the last Thursday of every month. *Pres.* J. B. Bean; *Sec.* S. M. Field.

*Massachusetts Dental Society*—Meets monthly. *Pres.* J. H. Batchelder, of Salem *MSec.* C. Wilson; *Cor. Sec.* L. D. Shepard.

M*chigan State Dental Association*—Meets annually second Tuesday of Oct., at Grand Rapids. *Pres.* T. R. Perry, Grand Rapids; *Rec. & Cor. Sec.* Wm. Tremper, Ypsilanti

*Mississippi Valley Dental Association* Meets annually at Cincinnati, on first Wednesday in March, 1876. *Pres.* George Watt, Xenia, Ohio; *Rec. Sec.* Wm. Taft, Cin.
*Mississippi State Dental Association* will meet 3d Wednesday of August. *Pres.* J. D. Miles; *Sec.* A. Riser.
*Minnesota Dental Associrtion* Meets annually. Next meeting at Minneapolis; May, 1876. *Pres.* F. A. Williamson, Reading, *Sec.* W. F. Lewis, Winona-St. Paul.
*Missouri Dental Association* Meets on the first Tuesday of June, in St. Louis. *Pres.* C. W. Rivers, St. Louis; *Sec.* W. H. Evans, St. Louis.
*Missouri Valley Dental Association* Meets on the fourth Tuesday in July, 1876, in Omaha, Neb. *Pres.* J. W. Chadduck; *Sec. and Treas.* F. M. Shrirer, Tabor, Iowa.
*Merrimac Valley Dental Association* Meets semi-annually first Tuesday of Oct. and May.* *Pres.* L. H. Kidder, Mass; *Rec. Sec.* A. M. Dudley, of Lowell; *Cor. Sec.* D. T. Porter, Lawrence, Mass.
*Memphis Dental Society*—Meets on the first Tuesday of each month. *Pres.* W. M. Arrington; *Sec.* V. F. Elliott; *Treas.* C. L. Harris.
*Nashville Dental ssociation*—Meets on the second Tuesday of each month. *Pres.* J. C. Ross; *Sec.* R. R. Freeman; *Cor. Sec.* S. J. Cobb.
*New Jersey State Dental Society*—Meets annually. Next meeting at Atlantic City, on the second Tuesday in July, 1876. *Pres.* C. S. Stockton, Newark; *Sec.* C. A. Meeker, Newark.
*New Haven Dental Society*—Meets on the first Wednesday of each month at New Haven. *Pres.* J. T. Metcalf, *Rec and Cor. Sec.* C. L. Smith, of New Haven.
*New Orleans Dental Society* Meets monthly. *Pres.* ———; *Rec. Sec.* A. F. McLane.
*Northern Ohio Dental Association.* Meets annually, second Tuesday of June, 1876, at Cleveland. *Pres.* E. J. Way, Sandusky; *Cor. Sec.* C. Buffett, Clinton.
*Northern Iowa Dental Association*—Meets annually, on the second Tuesday in June, 1873. Next meeting at Waterloo; *Pres.* J. N. Abbott, Lancaster, *Rec. Sec.* A. V Eaton, Anamosa; *Cor. Sec.* A. R. Mason, Waterloo.
*Ohio College Dental Association*—Meets annually Feb. 25. at Cincinnati. *Pres.* Jas. Leslie, Cin'ti.; *Rec. Sec.* H. A. Smith, of Cincinnati.
*Ohio State Dental Association*—Meets annually at Columbus. Next meeting, first Wednesday of December. *Pres.* C. R. Butler; *Rec. Sec.* F. A. Hunter, Cincinnati; *Cor. Sec.* C. R. Butler, Cleveland, O.
*Odontographic Society of Pennsylvania*—Meets on the first Wednesday of each month at 8 o'clock, P. M. in the Philadelphia Dental College. *Pres.* Louis Jack; *Cor. Sec.* J. H. McQuillen; *Rec. Sec.* E. L. Hewitt.
*Odontological Society of New York City* Meets the second Tuesday of each month. *Pres.* A. L. Northrup; *Rec. Sec.* Wm. Jarvie, Jr.
*Old Colony Dental Association* Meets quarterly.* *Pres.* C. G. Davis; *Rec. Sec.* L. W. Puffer, North Bridgewater, Mass; *Cor. Sec.* N. C. Fowler, Yarmouth, Mass.
*Ohio Valley Dental Association* Meets annually, in N. E. Ky; this year in Paris, Ky. Tuesday, Sept. 9th.
*Oregon State Dental Society* Meets annually. *Pres.* J. H. Hatch, Portland; *Rec. Sec.* J. R. Cardwell, Portland.
*Penn. Central Dental Asso.* meets on the 1st Tuesday of Nov. 1875, at Allentown. *Pres.* F. Hickman, *Sec.* R. E. Diefenderfer.
*Pennsylvania State Dental Society* meets next in Philadelphia, 2d Tuesday of July, 1876. *Pres.* E. T. Darby; *Cor. Sec.* M. H. Webb, Lancaster.
*Poughkeepsie Dental Society* Meets monthly. *Pres.* J. H. Mann; *Sec.* H. F. Clark
*Pennsylvania Association of Dental Surgery* Meets monthly in Philadelphia. *Pres* Spencer Roberts; *Sec.* E. R. Pettit.
*San Francisco Dental Association* Meets monthly. *Pres.* C. C. Knowles; *Cor. Se* H. E. Knox; *Rec. Sec.* Wm. Dutch.
*St. Louis Dental Society* Meets monthly. *Pres.* W. H. Morrison; *Sec.* A. H. Fuller.
*Susquehanna Dental Association* Meets annually.* *Pres.* C. S. Beck: *Rec. Sec.* W H. Nertz. Meets May, 1875, at Scranton.
*Society of Dental Surgeons of the City of New York* Meets semi-monthly in New York. *Pres.* B. F. Franklin: *Rec. Sec.* J. S. Latimer; *Cor. Sec.* E. H. Burras.
**Southern Dental Association* Meets annually. Next meeting at Memphis, Tenn., at time of Mardi-Gras. *Pres.* J. R. Walker, New Orleans, La.; *Rec. Sec.* J. F Thompson Va
*South Carolina State Dental Association* Meets annually. *Pres.* G. F. S. Wright, Columbia; *Sec.* F. T. Chupein, Charleston; *Cor. Sec.* J. S. Thompson, Abbeville. Next meeting at Greenville, S. C., second Thursday of June, 1876.
*Tennessee Dental Association* Meets semi-annually. Next meeting in Nashville, on the 4th Wednesday in June. 1876. *Pres.* R. R. Freema; *Rec. Sec.* H. W. Morgan, *Cor. Sec.* L. G. Noel.
*Virginia State Dental Association* Meets in Richmond, first Thursday in Nov. of each year. *Pres.* H. M. Grant, Abington: *Sec.* G. F. Kersee, Richmond.
*Washington, City Dental Society* Meets annually. *Pres.*, R. F. Hunt, *Sec.* Dr. Evans.
*Wabash Valley Dental Association* Meets annually. Next meeting at Wayne, third Tuesday in March, 1872. *Pres.* A. B. Cunningham, Attica: *Sec.* S. B. Brown, Fort Wayne.
*Wisconsin State Dental Association* Meets annually. Next meeting at Milwaukee, second Tuesday of July, 1876. *Pres.* R. S. Wells. Sparta; M. T. Moore, La Crosse.

### State and District Dental Societies of State of New York.

*New York State Dental Society*--Meets at Albany on the second Wednesday of July next. *Pres.* W. C. Barrett, Warsaw, N. Y.; *Cor. Sec.* S. B. Palmer; *Rec. Sec.* S. S. A, Freeman, Buffalo.

*First District* Meets second and fourth Wednesday evenings of each month. Annual meeting in New York, first Tuesday in June. *Pres.* J. S. Latiner New York *Rec. Sec.* F. M. Odell, New York.
*Second District.* Annual meeting in Brooklyn, first Monday in April. *Pres.* W. S. Elliott; *Rec. Sec.* M. E. Elmandorf. Brooklyn; *Cor. Sec.* F. W. Dalbare.
*Third District.* Meets semi-annually on second Tuesday in January and July. Annual meeting at Albany on second Tuesday in January. *Pres.* S. P. Welsh; *Rec. Sec.* H. A. Hall, Troy; *Cor. Sec.* J. A. Perkins, Albany.
*Fourth District.* Meets semi-annually. Meets annually at Saratoga on second Tuesday in June. *Pres.* C, A. Elmore, Ft. Edward; *Rec. Sec.* E. S. Pearsall, Saratoga.
*Fifth District.* Meets semi-annually. Annual meeting at Syracuse. second Tuesday in June. *Pres.* J. D. Huntington, Watertown; *Rec. Sec.* F. D. Nellis, Syracuse; *Cor. Sec.* J. S. Marshall, Syracuse.
*Sixth District.* Meets semi-annually. Annual meeting at Binghampton on second Tuesday in June. *Pres.* Frank B. Darby, Oswego; *Rec. Sec.* F. E. Perry, Norwich; *Cor. Sec.* A. M. Holmes, Morrisville.
*Seventh District.* Meets semi-annually. Annual meeting at Rochester first Tuesday in June. *Pres.* H. S. Miller; *Rec. Sec.* J. E. Line, Rochester.
*Eighth District.* Meets semi-annually. Annual on second Tuesday in May. *Pres* G. C. Daboll, *Rec. Sec.* S. A. Freeman, *Cor. Sec.* T. G. Lewis, Buffalo.
*The Seventh and Eighth District Societies* hold a union meeting on the last Tuesday of October. alternately at Buffalo and Rochester.

The Ohio State Board of Dental Examiners will meet in Columbus on the first Wednesday of Dec. 1875, at 10 o'clock. *Pres.* J. Taft; *Sec.* W. P. Horton.

### Exchanges.

ST. LOUIS MEDICAL AND SURGICAL JOURNAL.
BOSTON MEDICAL AND SURGICAL JOURNAL.
THE PACIFIC MEDICAL AND SURGICAL JOURNAL, SAN FRANCISCO.
PHIDADELPHIA MEDICAL AND SURGICAL JOURNAL.
CINCINNATI MEDICAL ADVANCE.
CINCINNATI LANCET AND OBSERVER.
DENTAL COSMOS, PHILADELPHIA.
LADIES' REPOSITORY, CINCINNATI.
HALL'S JOURNAL OF HEALTH, N. Y.
CHICAGO MEDICAL EXAMINER.
THE DETROIT REVIEW OF MEDICINE AND PHARMACY.
MEDICAL RECORD, N. Y.
NASHVILLE JOURNAL OF MEDICINE AND SURGERY.
AMERICAN JOURNAL OF DENTAL SCIENCE, BALTIMORE.
THE UNITED STATES MEDICAL AND SURGICAL JOURNAL, CHICAGO.
NEW ORLEANS JOURNAL OF MEDICINE.
AMERICAN AGRICULTURIST, N. Y.
BOSTON JOURNAL OF CHEMISTRY.
JOURNAL OF APPLIED CHEMISTRY, N. Y.
CANADA JOURNAL OF DENTAL SCIENCE, MONTREAL.
INDIANA JOURNAL OF MEDICINE.
MEDICAL AND SURGICAL REPORTER, SALEM, OREGON.
MISSOURI DENTAL JOURNAL, ST. LOUIS.
ECLECTIC MEDICAL JOURNAL, CINCINNATI.

### Ohio College of Dental Surgery.

The Thirtieth Annual Session of the Ohio College of Dental Surgery will begin on the 13th of October, 1875, and continue till March following.

FACULTY.

JAMES TAYLOR, M. D., D. D. S., Emeritus Institutes of Dental Science.
WM. CLENDENIN, M. D., Anatomy.
J. L. CILLEY, M. D., Physiology and Histology.
F. BRUNNING, M. D,, Pathology and Therapeutics.
J. TAFT, D. D. S., Operative Dentistry, Hygiene, Microscopy
J. S. CASSIDY, D. D. S., Chemistry.
J. I. TAYLOR, D. D. S., H. M. REID, D. D. S., Clinical Dentistry.
WM. VAN ANTWERP, D. D. S., Mechanical Dentistry.
J. L. CILLEY, M. D., Practical Anatomy.

| | |
|---|---|
| Matriculation Fee (but once) | $ 5 00 |
| Professors' Tickets for one session | 100 00 |
| Or for Winter and Spring terms | 130 00 |
| Demonstrator's Ticket (for Anatomy) | 5 00 |
| Diploma Fee | 30 00 |

TEXT BOOKS.

Gray's or Wilson's Anatomy; Dalton's, Carpenter's or Kirke's Physiology; U. S. Dispensatory; Fowne's, Turner's or Graham's Chemistry; Watt's Chemical Essays; Taft's Operative Dentistry; Richardson's Mechanical Dentistry; Harris' Principles and Practice of Dental Science; Tome's Dental Surgery; Harris' Dictionary of Dental Surgery.

TERMS OF GRADUATION.

A candidate for graduation must have two full years of pupilage, part of which, at least, should be with a reputable dental practitioner, and good teacher, inclusive of two complete courses of lectures in a Dental College.

A graduate of a respectable Medical College, who has had one year's pupilage under a reputable Dentist, or a student having had a regular pupilage of two years and four ye a practice, and otherwise satisfactory, may be admitted to the Senior Class, and to examination for the degree of D. D. S., after attending one full course of lectures.

For further particulars address the Dean or Secretary by letter.

Students coming to the city should call on the Dean. Good board can be had for $4 00 to 7 00 a week.

SPRING COURSE, 1876.

The Spring course in this Institution will commence on the 15th of March, and continue till the 1st of June.

Tickets for the course, $50, to be paid on entering, or $130 for Spring and Winter term.

J. TAFT, DEAN,

117 West 4th St.

F. BRUNNING, SEC.

THE POCKET

### DENTAL REGISTER.

Every dentist with anything like a full practice, and who cares to conduct business in a systematic manner, must have a book in which to record his dental operations.

The large "Registers" that have been used for that purpose, are excellent in their place, and may still be used in connection with such a book as this, if desired; but they are open to several important objections.

Many dentists using them have doubtless experienced the inconvenience of going to the desk after each patient's sitting, to make a record, and have preferred trusting to memory till after office hours. By that time, the exact tooth ope ated upon has often been forgotten; and sometimes a certain operation has slipped from the mind altogether.

The fillings put in to-day are marked on the same diagram with those inserted a month or a year ago; consequently, after a little while, it is impossible to distinguish between old and recent work.

The space reserved for each account being so large, one is unwilling to spoil a blank to record trifling services, such as extracting or treating a tooth for a patient whom he may never see again; although it often happens afterward that he would be glad to have a knowledge of the service.

In recording operations on the temporary teeth, it is very unsatisfactory to cross out the twelve back teeth of the permanent set, and imagine the bicuspids to be temporary molars. In such cases, much confusion generally follows in the course of a few years.

This Pocket Register is presented with the belief that it is free from these objectionable features.

Each operation may be recorded with pen or pencil, right at the chair, in almost the same time that an appointment is made for another sitting.

Every operation bears its own date, and cannot be taken for a previous or later one.

The space for accounts is so economically used, that one will not hesitate to record the most trivial services. It consists of 224 pages, arranged as follows:

120 pages for small account , ten to the page, with blanks for date, name, residence, operation, debit, and credit. Each account is also furnished with a diagram of the teeth for marking operations performed.
24 pages for medium accounts, two to the page, with blanks and diagram, as above.
16 pages for large accounts, one to the page, with blanks and diagram.
16 pages with blanks and diagrams expressly arranged for operations on the temporary teeth, ten accounts to the page.
32 pages for appointments for 64 weeks. These are without date, and may, therefore, be used for any time,
16 pages for index and memoranda.

It will be seen that the "Pocket Register" combines Account-book, Record of Operations on the Permanent and Temporary Teeth, Appointment-book, and Memoranda; and that the whole is offered for about the price of an ordinary Engagement book.

It is printed on fine writing paper, bound handsomely in leather, with tuck, or in English cloth, and is of a suitable size for carrying in the breast pocket.

—— PRICE. ——

*In Leather, with Tuck.* ........................................ $1 50
*In English Cloth,* ........................................ 1 25

Sent post-paid, on receipt of price. For sale, wholesale and retail, by

**For Sale by SPENCER, CROCKER & CO.**

### Ohio College of Dental Surgery.

The Thirtieth Annual Session of the Ohio College of Dental Surgery will begin on the 13th of October, 1875, and continue till the March following.

FACULTY.

JAMES TAYLOR, M. D., D. D. S., Emeritus Institutes of Dental Science.
WM. CLENDENIN, M. D., Anatomy.
J. L. CILLEY, M. D., Physiology and Histology.
F. BRUNNING, M. D., Pathology and Therapeutics.
J. TAFT, D. D. S., Operative Dentistry, Hygiene, Microscopy.
J. S. CASSIDY, D. D. S., Chemistry.
J. I. TAYLOR, D. D. S., H. M. REID, D. D. S., Clinical Dentistry
WM. VAN ANTWERP, D. D. S., Mechanical Dentistry.
J. L. CILLEY, M. D., Practical Anatomy.

| | |
|---|---|
| Matriculation Fee (but once) | $ 5 00 |
| Professors' Tickets for one session | 100 00 |
| Or for Winter and Spring terms | 130 00 |
| Demonstrator's Ticket (for Anatomy) | 5 00 |
| Diploma Fee | 30 00 |

TEXT BOOKS.

Gray's or Wilson's Anatomy; Dalton's, Carpenter's, or Kirke's Physiology; U. S. Dispensatory; Fowne's, Turner's or Graham's Chemistry; Watt's Chemical Essays; Taft's Operative Dentistry; Richardson's Mechanical Dentistry; Harris' Principles and Practice of Dental Science; Tome's Dental Surgery; Harris' Dictionary of Dental Surgery.

TERMS OF GRADUATION.

A candidate for graduation must have two full years of pupilage, part of which, at least, should be with a reputable dental practitioner, and good teacher, inclusive of two complete courses of lectures in a Dental College.

A graduate of a respectable Medical College, who has had one year's pupilage under a reputable Dentist, or a student having had a regular pupilage of two years and four years practice, and otherwise satisfactory, may be admitted to the Senior Class, and to examination for the degree of D. D. S., after attending one full course of lectures.

For further particulars address the Dean or Secretary by letter.

Students coming to the city should call on the Dean. Good board can be had for $4 00 to $7 00 a week.

SPRING COURSE, 1876.

The Spring course in this Institution will commence on the 15th of March, and continue till the 1st of June.

Tickets for the course, $50, to be paid on entering, or $130 for Spring and Winter term. J. TAFT, DEAN,
F. BRUNNING, SEC. 117 West 4th St.

